# Sex-specific expression and function of TRIM28 during mouse primordial germ differentiation

**DOI:** 10.1016/j.isci.2025.113474

**Published:** 2025-09-01

**Authors:** Jonathan A. DiRusso, Lingyu Zhan, Yu Tao, Allison L. Wang, Xinyu Xiang, Alexander C. Robbins, Azra J. Cruz, Wanlu Liu, Amander T. Clark

**Affiliations:** 1 Department of Molecular, Cell and Developmental Biology, University of California, Los Angeles, Los Angeles, CA 90095, USA; 2 Molecular Biology Institute, University of California, Los Angeles, Los Angeles, CA 90095, USA; 3 Eli and Edith Broad Center for Regenerative Medicine and Stem Cell Research, University of California, Los Angeles, Los Angeles, CA 90095, USA; 4 Center for Reproductive Science, Health and Education, University of California, Los Angeles, Los Angeles, CA 90095, USA; 5 Department of Psychiatry, University of California, Los Angeles, Los Angeles, CA 90095, USA; 6 Center for Neurobehavioral Genetics, Semel Institute for Neuroscience and Human Behavior, University of California, Los Angeles, Los Angeles, CA 90095, USA; 7 The Collaboratory, Institute for Quantitative and Computational Biosciences, University of California, Los Angeles, Los Angeles, CA 90095, USA; 8 Zhejiang University-University of Edinburgh Institute (ZJU-UoE Institute), Zhejiang University School of Medicine, International Campus, Zhejiang University, Haining 314400, China; 9 Department of Orthopedic of the Second Affiliated Hospital of Zhejiang University, School of Medicine, Zhejiang University, Hangzhou 310029, China; 10 Dr. Li Dak Sum & Yip Yio Chin Center for Stem Cell and Regenerative Medicine, Zhejiang University, Hangzhou 310058, Zhejiang, China; 11 Jonsson Comprehensive Cancer Center, University of California, Los Angeles, Los Angeles, CA 90095, USA

**Keywords:** Molecular biology, Epigenetics, Cell biology

## Abstract

Mammalian primordial germ cells (PGCs) are embryonic precursors to the adult germline and must facilitate high-fidelity transfer of genomic material from one generation to the next. Transposable elements (TEs) represent an ongoing threat to genomic fidelity and are therefore tightly controlled during embryonic germline development. Here, we find that some TEs change in accessibility during normal PGC differentiation, while others are constitutively repressed by tripartite motif-containing 28 (TRIM28), a master TE regulator. We find that TRIM28 itself is regulated in a sex-specific manner and represses sex-specific TEs. In both testicular and ovarian PGCs, TRIM28 protects against upregulation of 2-cell (2C)-associated genes, dysregulation of PGC differentiation, and incomplete activation of DAZL. This perturbs testicular and ovarian PGCs differently, with testicular PGCs failing to differentiate in embryonic life while ovarian PGCs inefficiently enter meiosis leading to a diminished ovarian reserve by the onset of sexual maturity.

## Highlights

• TRIM28 is responsible for repressing TEs during PGC differentiation
• Mutating TRIM28 in PGCs causes sex-specific differentiation outcomes
• Subfamilies of TEs are dynamically expressed during PGC differentiation
• Dynamically expressed TEs are not regulated by TRIM28

## Graphical abstract

**Figure:**
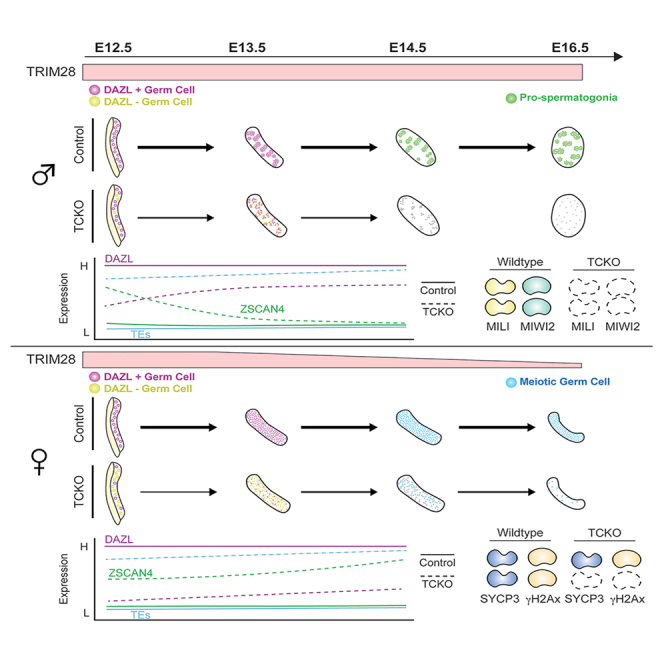

## Introduction

Sexual reproduction relies on the high-fidelity transfer of genetic information from one generation to the next. In sexually reproducing eukaryotes, genomic fidelity in the germline is antagonized by transposable elements (TEs), selfish genomic elements capable of transposition and expansion within the genome.^1^^,^^2^^,^^3^ TEs are relatively ubiquitous within eukaryotic genomes; only a few identified species lack them altogether.^4^^,^^5^ TEs are broadly classified as either type 1, retrotransposons, which move via an RNA intermediate, or type 2, DNA transposons, which move via a cut and paste mechanism.^6^^,^^7^ Uncontrolled movement of ether represents a potent threat to genomic integrity and organismal fitness, placing TE control in the germline under heavy selective pressure. Conversely, many TEs contribute positively to organismal fitness—a prominent example of this is in the mammalian placenta, which is reliant on syncytin genes derived from an endogenous retroviral *env* gene as well as retrotransposon-derived imprinted genes, which are also necessary for placental function.^8^^,^^9^ TEs also act as species-specific enhancers in the placenta, implicating them in multiple aspects of the evolution of placental mammals.^10^^,^^11^^,^^12^ In humans, a Hominidae-specific retrotransposon, LTR5Hs, acts as an enhancer during germline specification.^13^ As a result of this dichotomy, the germline and early embryonic cells are endowed with a rich network of highly conserved mechanisms to control deleterious TEs while enabling others to act beneficially.

The most thoroughly characterized TE mobility-control mechanisms in the germline of sexually reproducing organisms are those that utilize small RNA-mediated silencing. In the *Drosophila* ovary, germline cells utilize piwi interacting RNA (piRNA)s to silence TEs via an RNA-induced transcriptional silencing-like mechanism.^14^^,^^15^^,^^16^ A similar system is employed in pro-spermatogonial cells of the embryonic mouse testis, where piRNA-mediated silencing also drives targeted reacquisition of DNA methylation.^17^^,^^18^^,^^19^^,^^20^^,^^21^^,^^22^ TE control without piRNAs has been described as well. For example, the adult mouse oocytes utilize small interfering RNA (siRNA)-mediated TE degradation to repress evolutionarily young retrotransposons.^23^ While piRNA- and siRNA-mediated forms of repression act on the adult germline, how TE expression is regulated prior to sex determination in PGCs remains poorly characterized.

In sexually reproducing metazoans, germline development begins with specification of primordial germ cells (PGCs), embryonic precursors to the adult germline. In the mouse, specified PGCs acquire the expression of early PGC markers *Tfap2c*, *Prdm1* (*Blimp1*), and *Prdm14* as well as core pluripotency factors *Nanog*, *Sox2*, and *Oct4*.^24^^,^^25^^,^^26^^,^^27^^,^^28^^,^^29^ As PGCs migrate toward the genital ridges,^30^^,^^31^ they undergo genome-wide DNA demethylation and global re-arrangements of repressive epigenetic marks, including H3K9me3, H3K9me2, H3K27me3, and H2A/H4R3me2s.^32^^,^^33^^,^^34^^,^^35^^,^^36^ Following colonization of the gonad, PGCs upregulate DAZL, which is necessary to fully suppress the pre-sex determination PGC program and promote proper differentiation.^37^^,^^38^ In both testicular and ovarian PGCs, the timing of PGC differentiation relies on proper regulation of the PGC epigenome.

In mouse pluripotent stem cells and in early mouse embryos, the tripartite motif-containing 28 (*Trim28* a.k.a *Kap1*)/Krüppel-associated box domain zinc-finger protein (KRAB-ZFP) system is a major regulator of TE expression, particularly those of the long terminal repeat (LTR) retrotransposon subclass.^39^^,^^40^^,^^41^^,^^42^ When acting to repress LTRs, TRIM28 is targeted to sequence-specific sites in the genome by KRAB-ZFPs. Once bound, TRIM28 is then able to recruit the histone methyltransferase SETDB1, which catalyzes H3K9me3 at these sites to repress targeted loci.^43^ In PGCs, TEs are repressed by a diversity of mechanisms, including SETDB1-mediated H3K9me3, polycomb repressive complex 2 (PRC2)-mediated H3K27me3, and Dnmt1-mediated DNA methylation; however, the consequences of TE derepression are poorly defined.^44^^,^^45^^,^^46^^,^^47^

In the current study, we examine how control of TEs by *Trim28* safeguards the capacity of PGCs to differentiate into either primary oocytes or pro-spermatogonia. To do so, we used a conditional TRIM28 knockout (TCKO) mouse model to create a TRIM28 deletion in PGCs. We show that TRIM28 represses unique TE targets in both testicular and ovarian germ cells both directly and indirectly and that derepression of TEs is coupled with a misregulated transcriptome. Functionally, we show that TRIM28 loss results in improper differentiation, including defects in expression of DAZL and sex-specific defects in silencing the PGC program. We also show that ovarian and testicular PGC differentiation is differently sensitive to TRIM28 loss, with testicular PGCs failing to differentiate entirely while ovarian PGCs heterogeneously enter meiosis and show defects in meiotic progression and severe defects in the adult germline. Therefore, *Trim28* safeguards the germline by protecting the transcriptional program of PGCs as they undergo differentiation.

## Results

### TE expression is dynamic in PGCs

**Figure 1:**
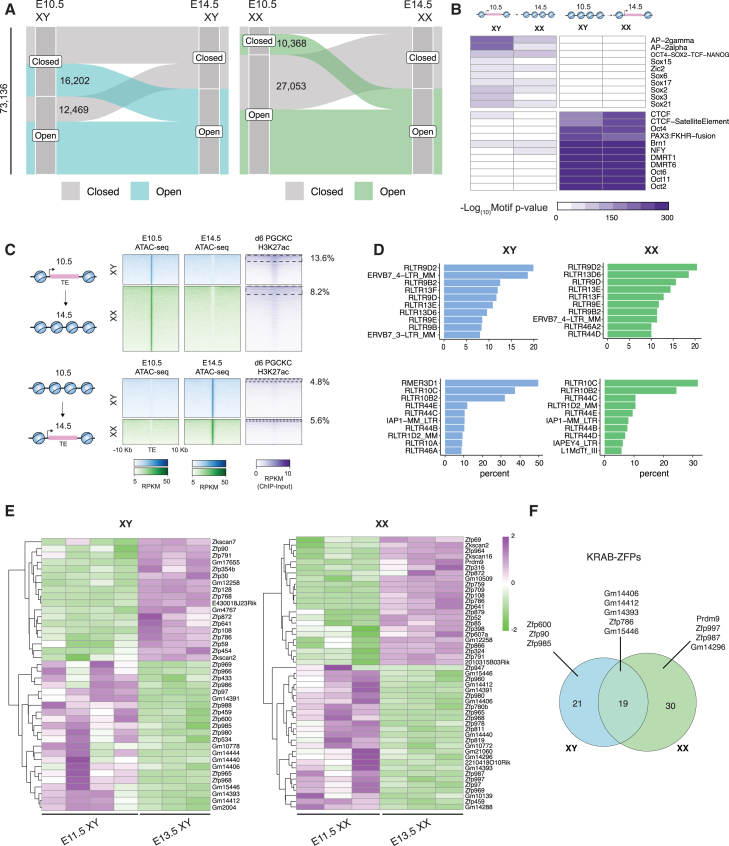
TE accessibility changes during PGC differentiation (A) Alluvial plots showing the chromatin accessibility state of TEs (ERV, LINE, and SINE) overlapping with ATAC peaks in XY (left) and XX (right). (B) Motif enrichment of TEs transitioning from OC (left) or CO (right) in either sex. *p* values from HOMER. (C) Chromatin accessibility of OC (top) or CO (bottom) TEs, enrichment of H3K27ac in *in vitro* PGCs. Percentages represent peak overlap. (D) Percentage of TEs within families for which the largest proportion of elements are represented. Top, TEs open to close; bottom, TEs close to open. (E) Heatmap showing differential gene expression between E11.5 and E13.5 testicular PGCs (left) and ovarian PGCs (right). (F) Venn diagram showing sex-specific and shared KRAB-ZFPs. See also Figure S1; Table S5.

To understand how TEs are regulated as PGCs differentiate, we first set out to characterize the changes in TE accessibility in the mouse as PGCs advance from migratory PGCs to differentiated germ cells. To do so, we used assay for transposase accessible chromatin - sequencing (ATAC-seq) from PGCs collected via fluorescence-activated cell sorting (FACS) using an Oct4-eGFP reporter at E10.5, which is when PGCs have entered the sexually indifferent genital ridge, and E14.5, at which point the chromatin accessibility landscape reflects differentiated germ cells in the ovary or testis.^48^ From both XY and XX samples, we identified 73,136 TEs across the endogenous retrovirus (ERV), long interspersed nuclear element (LINE), and short interspersed nuclear element (SINE) superfamilies, which are accessible in at least one condition. We then identified elements that were accessible at E10.5 but not E14.5 (open to closed [OC], 12,469 in XY; 27,053 in XX) or those that were accessible at E14.5 but not E10.5 (closed to open [CO], 16,202 in XY; 10,368 in XX). (Figure 1A). As we found more accessible TEs in XX PGCs than XY PGC at E10.5, we wanted to rule out the possibility that X chromosome dynamics drove this observation. To do so, we compared the number of accessible TEs detected per chromosome to the total number of TEs on that chromosome (Figure S1A). We did not find a significant increase in the ratio detected in the X chromosome compared to autosomes, ruling out X chromosome dynamics as a significant driver of this observation. Therefore, even prior to acquiring a sex-specific developmental program, XX and XY PGCs have different TE repertoires.

We next moved to characterize differentially accessible TEs, which transitioned from OC or CO to better understand how these TEs could be embedded into the *cis*-regulatory network of PGCs. To analyze this, we performed motif enrichment analysis on identified TEs (Figure 1B). Consistent with possible *cis*-regulatory roles for these TEs, we found motifs for AP2-gamma and AP2-alpha (TFAP2C and TFAP2A, respectively), as well as the OCT-SOX-NANOG and Zic2 motifs, to be significantly enriched in TEs, which progress from OC. To further examine whether these TEs may have functional relevance, we examined the enrichment of H3K27ac, a marker of active enhancers and promoters. To do so, we used published data from PGC-like cells, an *in vitro* PGC model, which recapitulates ∼ E10.5 PGCs *in vivo.*^49^ We found that H3K27ac peaks overlapped at 13.6% of OC TEs in XY PGCs and in 8.2% of OC TEs in XX PGCs across ERVs, LINEs, and SINEs (Figure 1C). In line with these observations, we found pluripotency-associated subfamilies RLTR13D6 and RLTR9E to be enriched, with ∼10% of RLTR13D6 elements transitioning from OC in XY and 18% in XX PGCs.^50^^,^^51^ For RLTR9E, ∼10% of all elements transitioned from OC in both XY and XX PGCs (Figure 1D). In both sexes, the top enriched subfamily was RLTR9D2, which has not been functionally evaluated but is enriched for H3K27ac and H3K4me1 in mouse embryonic stem cells (mESCs).^52^ Thus, it is possible that proper silencing of these families marks progression from PGC identity to differentiated germ cell.

Conversely, TEs that transition from closed at E10.5 to open at E14.5 (CO) were enriched for DMRT1, DMRT6, and CTCF motifs in both testicular and ovarian germ cells (Figure 1B). DMRT1 has been implicated in germline differentiation and DMRT6 in maintenance of the pro-spermatogonial transcriptional program.^53^ As expected, these TEs had little enrichment for H3K27ac in day 6 PGC-like cells, which would agree with these TEs being associated with differentiated germ cells (Figure 1C). Among CO differentially accessible TEs, RLTR10C and RLTR10B2 were enriched in both testicular and ovarian germ cells. Of interest is RLTR10B2, which has been functionally validated as an enhancer necessary for the mitosis-to-meiosis transition in spermatocytes.^54^ We also found enrichment of RLTR10C, which is upregulated in spermatocytes as well, but any *cis*-regulatory function has not been mechanistically assessed (Figure 1D). When we analyzed differentially accessible TEs for H3K27ac enrichment using previous published chromatin immunoprecipitation sequencing (ChIP-seq) from testicular and ovarian PGCs at E13.5, we found that only 4.3% of H3K27ac peaks overlapped with TEs, which transitioned from CO in testicular PGCs and 1.9% in ovarian germ cells (Figure S1D). Thus, while the TEs which become accessible are associated with later-stage function by motif enrichment, they do not appear to acquire enhancer-like properties at either E10.5 or E13.5, as marked by acquisition of H3K27ac.

We next asked whether large changes in TE accessibility during PGC development were a phenomenon specific to the germline or if a similar change is found in the gonadal soma, which also undergoes rapid development and differentiation during this window. To do so, we used published ATAC-seq data of gonadal soma from E10.5 and E13.5.^55^ Like PGCs, we found that testicular and ovarian soma had unique TE dynamics (Figure S1E). A motif analysis found that TEs which are open at E10.5 and close by E13.5 are enriched for Hoxc9 and Hoxa9, while those that become accessible by E13.5 are enriched for Nr5a2, Dmrt1/6, and COUPTFII (Nr2f2) motifs (Figure S1F). Thus, large changes in TE repertoire may be a conserved property of gonadal development.

Given that ERVs are known to harbor transcription factor motifs, we hypothesized that a driver of some of these changes in the chromatin accessibility of TEs may be TRIM28. TRIM28 is an epigenetic scaffolding protein recruited to loci by KRAB-ZFPs and in turn coordinates with NuRD and SETDB1 to catalyze repressive heterochromatin at targeted loci. We hypothesized that our observed shift in TE accessibility as PGCs progress from E10.5 to E14.5 should also be marked with a concomitant change in KRAB-ZFP repertoire. To test this, we performed RNA sequencing (RNA-seq) on both testicular and ovarian PGCs at E11.5, E12.5, and E13.5. We compared differential expression of KRAB-ZFPs between E11.5 and E13.5, identifying 40 differentially expressed (DE) KRAB-ZFPs in XY PGCs between E11.5 and E13.5 and 49 DE KRAB-ZFPs in XX PGCs (Figures 1E and 1F). Of those, 19 were shared by both XY and XX PGCs (Figure S1G). Of those only in XY PGCs, *Zfp985* and *Zfp59* have both been identified in later-stage male germline development, including pachytene spermatocytes and round spermatid, respectively. In females, *Prdm9* is a known regulator of double-strand breaks during meiosis, while *Zfp987* has been characterized as a repressor of LTR family elements.^56^^,^^57^ Finally, we found KRAB-ZFPs, which targeted LINEs (Gm14406 and Gm14412), and endogenous retrovirus element K (ERVK) family (Gm15446) elements to be DE in both sexes as PGCs differentiate.^57^ Therefore, the transition between early and differentiated PGCs is marked by large-scale changes to TE accessibility and, contemporaneously, changes to the repertoire of KRAB-ZFPs.

### TRIM28 regulation of TEs is sex specific

**Figure 2:**
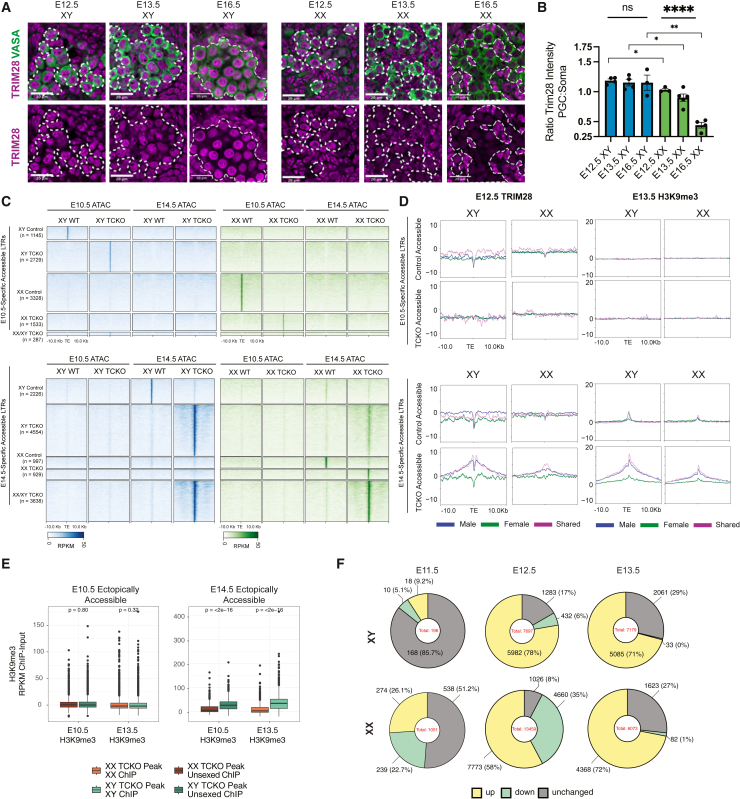
TRIM28 repression of TEs is sex specific (A) Immunofluorescence showing TRIM28 at E12.5, E13.5, and E16.5 in XY (left) and XX (right) samples. Germ cells are marked with VASA (green) and TRIM28 in magenta. Scale bars represent 20 μm. *N* ≥ 3 for all conditions. (B) Quantification of TRIM28 abundance in PGCs relative to gonadal soma. Each dot represents a biological replicate. Pairwise comparisons are t tests, and three-way comparisons are ANOVA tests. Error bars in SEM. (C) Heatmaps showing ERV accessibility in control and TRIM28 knockout PGCs at E10.5 and E14.5. (D) Profile plots of ChIP-seq data of TRIM28 at E12.5 (left) or H3K9me3 at E13.5 (right) at ERVs accessible in control PGCs (top) or TCKO PGCs (bottom). Graphs show input-subtracted normalized to read count. (E) H3K9me3 enrichment at LTRs identified at E10.5 (left), E14.5 (right), and E13.5. Significance tested using unpaired 2-sided t test. Boxplot shows median with hinges equal to 25th and 75th percentiles, whiskers are 1.5 interquartile range. (F) Proportional representation of TEs, which are upregulated (false discovery rate [FDR] < 0.005, Log2FC > 2), downregulated (FDR < 0.005, Log2FC < 0.25), or unchanged (log2fold change 0.99 to 1.005, FDR which can be calculated). TEs that did not meet these criteria are not plotted. As a result, the total number of analyzed TEs varies between developmental stages and sexes. Total number of assessed TEs in middle of chart. Number of DETEs and percentage of TEs matching criteria are shown. For statistical tests, ns = *p* > 0.05, ∗*p* < 0.05, ∗∗*p* < 0.01, ∗∗∗*p* < 0.001, ∗∗∗∗*p* < 0.0001. See also Figure S2; Tables S6 and S7.

We began by characterizing the expression of TRIM28 in PGCs as they differentiate. To do so, we assessed the abundance of TRIM28 at E12.5, E13.5, and E16.5. We noticed that the abundance of TRIM28 decreased in ovarian germ cells between E12.5 and E16.5 (Figure 2A). To better quantify this, we compared the abundance of TRIM28 in germ cells to that of the surrounding gonadal soma. In testicular germ cells, we found the relative abundance of TRIM28 to be consistent at each time point. The relative abundance of TRIM28 in ovarian germ cells decreased significantly and qualitatively between E12.5 and E16.5 (Figure 2B). Thus, TRIM28 is regulated in a sex-specific manner in PGCs as they differentiate, in line with sex-specific changes to TE accessibility. Based on these results, we wondered whether the function of TRIM28 is sex specific as PGCs differentiate.

To better understand which TEs TRIM28 was regulating and if there were sex-specific differences in TRIM28 activity, we performed ChIP-seq of TRIM28 at E12.5, as PGCs are initiating differentiation. Consistent with our findings showing lower TRIM28 abundance in ovarian PGCs, ChIP-seq identified fewer TRIM28-binding sites in ovarian PGCs (1,406 peaks) compared to testicular PGCs (5,689 peaks). Interestingly, we found little enrichment of TRIM28 at E12.5 or H3K9me3 at E13.5^45^ over the dynamic TEs that progress from open at E10.5 to closed at E14.5 (Figure S2A), suggesting that TRIM28 does not act directly at the dynamic loci in the germline at this stage and instead is likely acting at the sites that are constitutively closed.

To identify TEs that are regulated by TRIM28, we conditionally mutated *Trim28* in PGCs using *Blimp1-Cre*. We refer to this as a TCKO as TRIM28 is absent in the PGCs but not soma (Figures S2B and S2C). In addition, we bred an *Oct4-eGFP* reporter allele into the strain to enable FACS isolation of PGCs at E10.5 through P1, covering the window during which the repertoire of accessible TEs in the germline changes rapidly^58^ (Figure 1A). As *Blimp1* (*Prmd1*) is expressed in the placenta as well, we also assessed embryo weight at E14.5 to ensure there were no gross developmental defects or signs of delayed embryo growth. We found no difference in weight or appearance at E14.5 (Figure S2D), suggesting that gross developmental dynamics are unaffected by *Blimp1-cre*-induced knockout of *Trim28* between PGC specification (E6.25) and differentiation (E14.5).

TRIM28 is a well-known regulator of ERVs; therefore, we focused our analysis only on ERVs. In agreement with our initial analysis, we identified more accessible ERVs in XX PGCs than their XY counterparts at E10.5 and found that both XY and XX PGCs had distinct subsets of accessible ERVs in the absence of TRIM28, which may be due to indirect effects of TRIM28 (Figure 2C). In agreement with this, these ectopically accessible ERVs were not bound by TRIM28 and showed no H3K9me3 enrichment (Figure 2D). Importantly, we found them to be closed successfully in the absence of TRIM28 and therefore are not direct targets of TRIM28. We found this to be true for ERVs that are accessible in control conditions as well, in agreement with our observation that changes in TE accessibility in PGCs are independent of TRIM28 function prior to E12.5.

In contrast to E10.5 PGCs, we discovered 3,638 ERV loci which were ectopically accessible in both testicular and ovarian TCKO PGCs (Figure 2C). Ectopically accessible ERVs identified only in testicular germ cells as well as those shared with both testicular and ovarian germ cells were enriched for TRIM28 at E12.5 and H3K9me3 at E10.5 and E13.5, indicating that this subset of ERVs is likely directly regulated by TRIM28 and is constitutively silenced during PGC differentiation (Figure 2E). Ectopically accessible ERVs that are unique to ovarian germ cells at E14.5 were not bound by TRIM28 in control PGCs and were not enriched in H3K9me3 (Figures 2D and S2E). Taken together, these results demonstrate that TRIM28 plays a direct role in regulating a shared set of ERVs in both ovarian and testicular PGCs as they gain competence to differentiate, while some ERVs in ovarian PGCs are indirectly affected by a knockout of TRIM28 and are silenced independently of TRIM28. This sexual dimorphism in the control of ERVs aligns with a reduced TRIM28 abundance in ovarian PGCs as they differentiate, where other mechanisms of TE control likely act at these loci.

As ERVs directly repressed by TRIM28 were found to be ectopically accessible at E14.5 in the TCKO PGCs, we asked at which point do ERVs become derepressed in the TCKO PGCs. Comparing expression of ERVs in control and TCKO PGCs via RNA-seq, we find that ERVs are rapidly derepressed between E11.5 and E12.5 (Figure 2F). Starting at E12.5, both testicular and ovarian PCGs showed robust derepression of ERVs, with the largest proportional derepression in intracisternal A particle (IAP) subfamilies (Figure S2G). These ERVs are evolutionarily young and expected to be under robust control by TRIM28. Between ovarian and testicular PGCs, the dynamics of derepression did differ slightly. In ovarian PGCs at E12.5, 58% of detectable LTRs were derepressed whereas 35% had significantly reduced expression relative to controls. In contrast, 77% of detectable LTRs in testicular PGCs were derepressed and only 6% were significantly decreased (Figure 2F). While it is unclear how loss of TRIM28 would lead to a reduction in TE RNA, this result does demonstrate differences in response to loss of TRIM28 between ovarian and testicular PGCs. Our results here show that loss of TRIM28 drives major TE derepression at E12.5, a critical time point during which PGCs are sensitive to perturbation of the epigenome. We also identified some increases in TE expression in the absence of TRIM28, which were more abundant in XX PGCs and may be due to indirect effects on TE regulation.

### Loss of TRIM28 affects gene expression in PGCs and induction of 2C-associated genes

**Figure 3:**
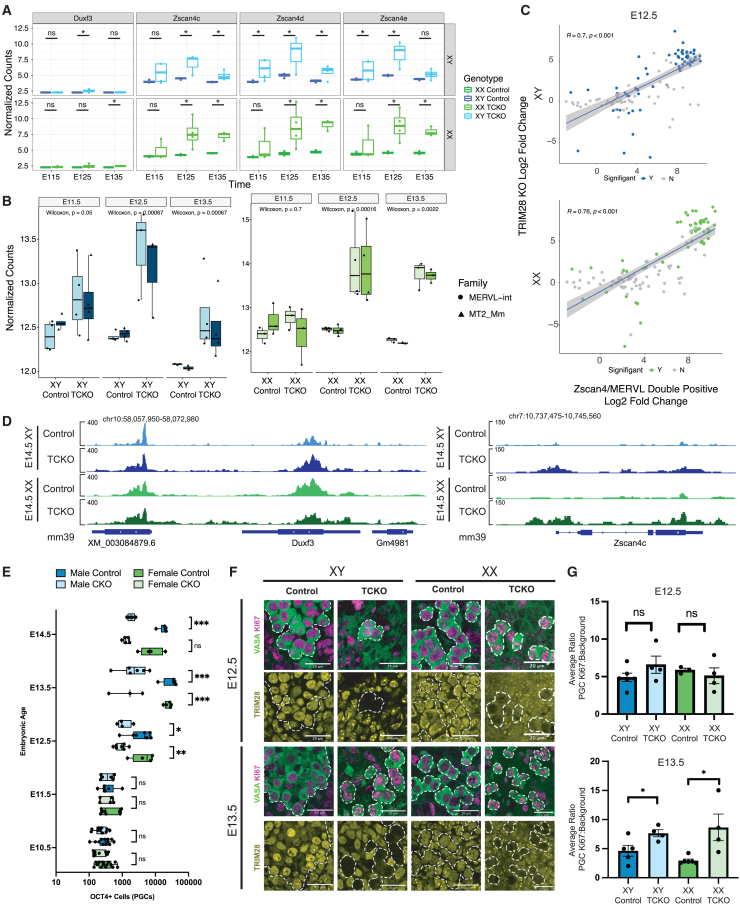
TRIM28 suppresses emergence of a 2C-like transcriptome (A) Expression of select genes in E11.5, E12.5, and E13.5 control and TCKO PGCs in XY (top) and XX (bottom). Significance by DESeq2 (log2 fold change ≥ 2 or ≤ −2 and *p*adj. < 0.05). (B) Normalized RNA-seq of MERVL-int and MT2_Mm at E11.5, E12.5, and E13.5 in control and TCKO PGCs. *p* value is Wilcoxon t test. (C) Correlation of Log2 fold change 2C transcriptome genes between mESCs/2CLCs (*x* axis) and control/TCKO PGCs (*y* axis) at E12.5. Colored dots are significant in TRIM28 DEG analysis. (D) Browser track plots of ATAC-seq at E14.5 in control and TCKO XY (top) and XX (bottom) PGCs at the *Dux* locus (left) and *Zscan4c* (right). RPKM normalized. (E) Boxplot of Oct4-eGFP^+^ PGCs isolated at indicated time point. Significance testing by t test. (F) Representative images of Ki67 and TRIM28 staining in testicular (XY) and ovarian (XX) PGCs. VASA marks germ cells. Scale bars represent 20 μm. (G) Quantification of background-corrected Ki67 intensity in E12.5 (top) and E13.5 (bottom) testicular and ovarian PGCs. Average ratio of each biological replicate. Significance testing by Welch’s t test. Error bars in SEM. At least, *n* = 3 biological samples. For statistical tests, ns = *p* > 0.05, ∗*p* < 0.05, ∗∗*p* < 0.01, ∗∗∗*p* < 0.001, ∗∗∗∗*p* < 0.0001. See also Figure S3; Tables S7 and S8.

In mESCs, loss of TRIM28 is associated with a disrupted transcriptome ultimately leading to loss of self-renewal.^39^ Here, we evaluated whether a similar phenomenon might be occurring in TCKO PGCs. To identify differentially expressed genes (DEGs) in TCKO PGCs compared to controls, we compared the RNA-seq datasets of TCKO and control PGCs at E11.5, E12.5, and E13.5. We found a statistically significant increase in the expression of genes in TCKO PGCs, which are associated with the 2-cell (2C) stage embryo, including *Duxf3* and *Zscan4* (Figure 3A). We also found upregulation of MERVL-int and its associated LTR, MT2a_Mm (Figure 3B), both of which are also highly expressed in the 2C mouse embryo. Misregulation of the 2C-associated genes and ERVs had sex-specific patterns. In testicular PGCs, *Duxf3*, *Zscan4*, MERVL-int, and MT2a_Mm genes were highly upregulated by E12.5 but fell by E13.5, while in ovarian PGCs, they were upregulated at E12.5 but remained highly upregulated at E13.5 (Figures 3A and 3B). In both sexes, we found expression of these genes to be highly variable, suggesting that their expression may be heterogeneous within TCKO PGCs. We next performed Gene Ontology (GO)-enriched gene set enrichment analysis at E12.5, when the phenotype first emerges. In testicular PGCs, the top enriched terms included repression of genes related to rRNA production and biogenesis, while in ovarian PGCs, DNA replication, repair, and histone modification were likewise repressed terms (Figure S3A). As disruption to ribosome biogenesis and cell-cycle progression has both been demonstrated to induce a 2C-like state, we wondered whether TCKO PGCs more broadly acquired a 2C-like transcriptome.^59^^,^^60^

To assess this, we compared changes in 2C-associated gene expression in TCKO PGCs to the expression of these genes in 2C-like cells (2CLCs).^61^ We found that TCKO PGCs at E12.5 and E13.5 had a robust correlation with 2CLC-associated genes (Figures 3C and S3C). However, the transcriptome of TCKO PGCs does not cluster with 2CLCs in a principal-component analysis, confirming that TCKO PGCs and 2CLCs remain transcriptomically distinct (Figure S3B). We next tested whether ectopically accessible ERVs could be acting as enhancers for the upregulated 2C-associated genes. To test this, we used the RAD algorithm^62^ and compared ectopically accessible ERVs at E14.5 in each sex to DEGs. This result shows that ectopically accessible ERVs are statistically associated with upregulated distal 2C genes (greater than 5 to 25 kb up- or downstream) (Figure S3D). Taken together, we propose that the increased expression of 2C-associated genes in TCKO PGCs is likely influenced by neighboring changes in accessibility of ERVs. However, PGCs are not acquiring a 2CLC transcriptional state.

During exit from the 2C state, TRIM28 is thought to pair with *Line1* RNAs to promote silencing of *Dux*, after which embryonic development can progress.^63^^,^^64^ Given that *Duxf3* expression was increased in TCKO PGCs, we evaluated the chromatin state of *Duxf3* and found it to be accessible in both control and TCKO PGCs (Figure 3D). Conversely, *Zscan4c* gains accessibility in TCKO PGCs (Figure 3D). To confirm that *Duxf3* is normally accessible in E14.5 germ cells, we also examined accessibility in a published DNase sequencing dataset^48^ and found the same result (Figure S3F). As *Zscan4c* has been shown to promote 2C-like expression, we next asked if a putative *Zscan4* enhancer previously shown to be sensitive to derepression by loss of TRIM28 was bound by TRIM28 in PGCs.^65^^,^^66^ Indeed, we found TRIM28 and H3K9me3 enriched at the putative enhancer (Figure S3E). Taken together, TRIM28 loss in PGCs results in the emergence of 2C-associated genes likely due to convergence of neighboring ERVs that become accessible in the absence of TRIM28 combined with derepression of TRIM28-targeted *Zscan4* enhancer.

Given that PGCs enter a rapid mitosis between E11.5 and E13.5,^67^^,^^68^ and this is when the major increase in TE expression occurs, we asked whether there is a difference in the number of TCKO PGCs during this window. We found a significant decrease in PGC number at E12.5 and E13.5 (Figure 3E). To test whether TCKO PGCs at E12.5 and E13.5 were in cycle, we performed immunofluorescence for Ki67, which marks cells outside of prolonged G_0_ or G_1_.^69^ At E13.5, both testicular and ovarian TCKO PGCs had significantly higher abundances of Ki67 compared to controls (Figures 3F and 3G). TCKO PGCs between E12.5 and E13.5 fail to increase in number as rapidly as control PGCs (Figure 3E) yet retain higher Ki67 abundance at E13.5, which may imply that TCKO PGCs progress through the cell cycle more slowly and, therefore, remain in the cycle longer. To ensure that PGCs were not undergoing apoptosis or acquiring double-stranded DNA breaks (DSBs), we examined the abundance of cPARP, a marker of apoptosis, and yH2Ax, a marker of DSBs. We found no evidence of widespread DSBs or evidence of apoptosis (Figures S3G–S3I), further supporting delayed cell cycle progression in TCKO PGCs as the rationale for lower PGC numbers in TCKOs compared to controls.

Taken together, our results show that TRIM28 loss is associated with expression of 2C-associated genes at E12.5 and this is associated with significantly fewer PGCs in the ovary and testis compared to controls.

### TRIM28 is required for PGC competency

**Figure 4:**
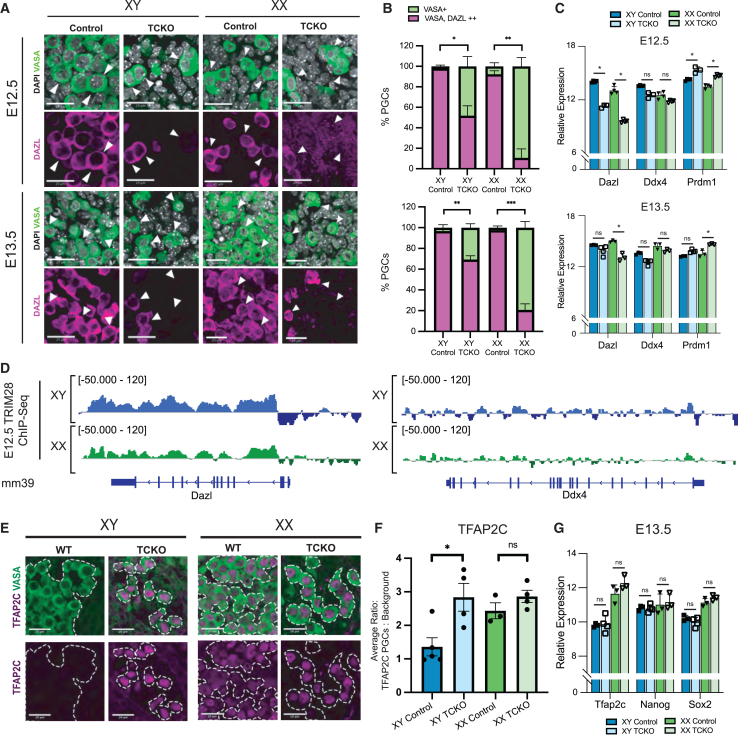
TRIM28 is required for timely acquisition of competency (A) Representative images at E12.5 and E13.5 showing PGCs (VASA) and DAZL. Scale bars represent 20 μm. (B) Quantification of percentage of PGCs positive for DAZL and VASA or only VASA in testicular (XY) and ovarian (XX) PGCs at E12.5 (top) (XX control *n* = 4, XX TCKO *n* = 3, XY control *n* = 3, XY TCKO *n* = 3) and E13.5 (bottom) (XX control *n* = 4, XX TCKO *n* = 4, XY control *n* = 3, XY TCKO *n* = 3). Significance testing by Welch’s t test. Error bars in SEM. (C) Normalized expression of *Dazl*, *Ddx4*, and *Prdm1*. Error bars in SD. (D) Gene tracks showing abundance of TRIM28 over the *Dazl* locus (left) and *Ddx4* locus (right) at E12.5. (E) Representative images of embryonic testes and ovaries with TFAP2C and VASA. Scale bars represent 20 μm. (F) Quantification of TFAP2C intensity over background in PGCs, each dot represents the mean of one biological replicate. Significance by Welch’s t test. Error bars in SEM. (G) Normalized expression of *Tfap2c*, *Nanog*, and *Sox2* in E13.5 PGCs. Error bars in SD. For t tests, ns = *p* > 0.05, ∗*p* < 0.05, ∗∗*p* < 0.01, ∗∗∗*p* < 0.001, ∗∗∗*p* < 0.0001. See also Figure S4.

Next, we evaluated the germline competency marker *Dazl*. At E12.5, nearly all control PGCs are DAZL^+^; however, we identified sex-specific differences in DAZL expression in TCKO mutants with 10.71% of ovarian and 51.83% of testicular TCKO PGCs being positive for DAZL at E12.5 (Figures 4A and 4B). At E13.5, DAZL is still negative in a large fraction of TCKO PGCs again with sex-specific differences, with TCKO ovarian PGCs having a greater defect in DAZL expression than testicular PGCs (Figures 4A and 4B). To evaluate whether defects in DAZL expression were the result of transcriptional or post-transcriptional misregulation, we examined the RNA-seq data and discovered significantly reduced levels of *Dazl* at E12.5 (Figure 4C). An alternate gonadal PGC gene *Ddx4* (*Vasa*) was not differently expressed. At E13.5, *Dazl* was significantly reduced in ovarian but not testicular TCKO germ cells, aligning with a more severe reduction in DAZL-positive PGCs in the ovary compared to PGCs in the testis at E13.5 (Figures 4B and 4C). As our transcriptomic results mirrored the dynamics of DAZL observed via immunofluorescence, we reasoned that the reduction in DAZL^+^ PGCs is likely due to differences in *Dazl* transcription, rather than by post-transcriptional or translational control.

We next asked whether this phenotype could be a direct effect of TRIM28. Analysis of TRIM28 ChIP-seq revealed that the *Dazl* gene body is enriched in TRIM28 at E12.5 (Figure 4D). In contrast, the gene bodies of other expressed germline genes, *Ddx4* (Vasa) and *Prdm1*, were not enriched in TRIM28 (Figures 4D and S4A). Therefore, it is possible that TRIM28 has some direct action on the DAZL locus itself, although it is unclear what role TRIM28 may have, and our data suggest that this role is unlikely to be repressive.

Given that a null mutation in *Dazl* leads to persistence of pluripotent gene expression in the germline,^70^ we examined the abundance of pluripotency proteins NANOG, SOX2, and TFAP2C (AP2-gamma) using immunofluorescence. SOX2 and NANOG were appropriately downregulated between E12.5 and E13.5 in TCKO PGCs (Figures S4B and S4D; Figures S4C and S4E). In contrast, TFAP2C protein (Figures 4E and 4F) but not RNA exhibited higher abundance in TCKO testicular PGCs at E13.5 (Figure 4G). At E14.5, we found that some ovarian TCKO germ cells remain NANOG or SOX2 positive (Figure S4F). In testicular germ cells, we found control testicular germ cells to be partially positive for NANOG and SOX2 at E14.5 and therefore examined E16.5 pro-spermatogonia for NANOG or SOX2 and found it to be absent both in controls and TCKO germ cells (Figure S4G). Therefore, a deletion of TRIM28 is associated with inefficient silencing of some early PGC-expressed proteins, as testicular TCKO PGCs fail to efficiently eliminate TFAP2C and ovarian PGCs fail to fully eliminate SOX2 and NANOG despite transcriptomic downregulation. Together, these results demonstrate that TRIM28 is required for proper DAZL induction and timely early PGC protein repression.

### TRIM28 has sex-specific effects on germ cell differentiation

We next evaluated germ cell differentiation at E16.5. To evaluate germ cell differentiation in the testis, we analyzed two pro-spermatogonia genes, MILI (*Piwil2*) and MIWI2 (*Piwil4*). For germ cell differentiation in the ovary, we evaluated γH2Ax (H2AXpS139) and synaptonemal complex protein 3.

**Figure 5:**
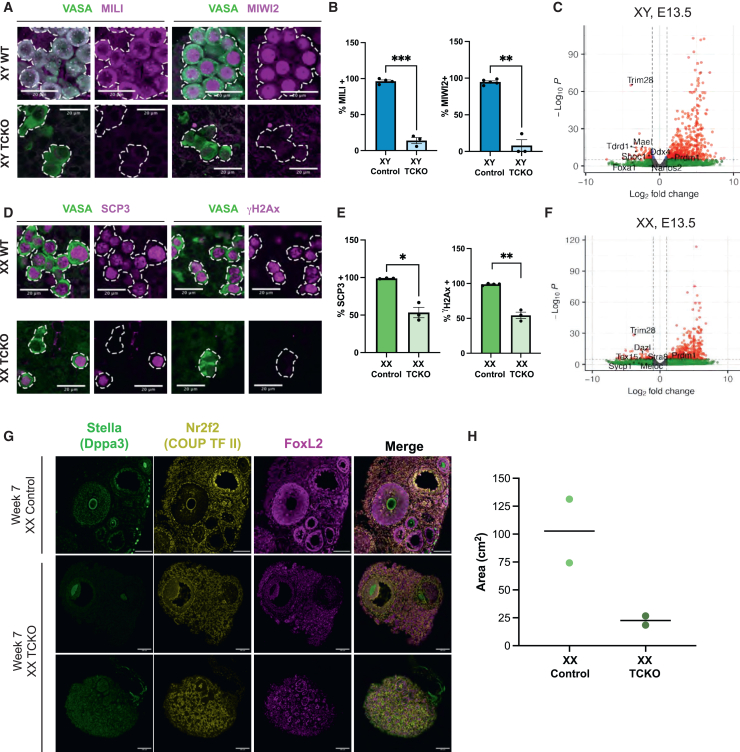
TRIM28 is necessary for successful gametogenesis (A) Representative image of embryonic testes showing MILI (left) and MIWI2 (right) in pro-spermatogonia (VASA). Scale bars represent 20 μm. (B) Quantification of percentage of MILI^+^ (left) and MIWI2^+^ (right). Each point is one biological replicate. Significance by Welch’s t test. Error bars show SEM. (C) Volcano plot showing changes in expression in E13.5 testicular PGCs. Select genes are labeled. (D) Representative image of embryonic ovaries showing expression of SCP3 (left) and yH2Ax (right) in meiotic germ cells (VASA). Scale bars represent 20 μm. (E) Quantification of percent SCP3^+^ (left) and yH2Ax^+^ (right) in E16.5 ovaries. Each point is one biological replicate. Significance by Welch’s t test. Error bars show SEM. (F) Volcano plot showing changes in expression in E13.5 testicular PGCs. Select genes labeled. (G) Immunofluorescence images of week 7 (day 48) ovaries from 1 control female and 1 TCKO female. Ovarian soma (NR2F2), granulosa cells (FOXL2), and oocyte (DPPA3) are shown. Each TCKO ovary is shown. Scale bars represent 100 μm. (H) Measurement of ovary area from 2D image in cm^2^. Each dot is a single ovary from one biological replicate. For t tests, ns = *p* > 0.05, ∗*p* < 0.05, ∗∗*p* < 0.01, ∗∗∗*p* < 0.001, ∗∗∗∗*p* < 0.0001. See also Figure S5.

Consistent with abnormalities in germ cell differentiation, TCKO germ cells in the testis at E16.5 failed to express MILI and MIWI2, whereas nearly all control pro-spermatogonia expressed both proteins with the expected subcellular localization (Figures 5A and 5B).^71^ VASA also failed to show the distinct punctate appearance indicative of co-localization with MILI, as observed in controls. RNA-seq data from E13.5 testicular PGCs show that other members of the piRNA pathway, including *Tdrd1* and *Mael*, had significantly reduced expression as well (Figure 5C). Additionally, a GO analysis of significantly downregulated genes in TCKO PGCs at E13.5 was enriched for GO terms associated with developmental processes (GO 0032502, 0009888, 0050793) (Figures S5A and S5B). Testicular germ-cell-specific genes such as *Nanos2* were not significantly different. Thus, while TCKO PGCs in the testis failed to differentiate by E16.5, they do still initiate aspects of the male germline program. We also examined a P1 TCKO knockout and found VASA^+^ germ cells to be rare. While these germ cells did express MIWI2, expression was weak and lacked the distinct localization typical of MIWI2 (Figure S5F). Given these results, we conclude that TRIM28 knockout results in disordered differentiation and ultimately loss of the male germline.

In embryonic ovaries at E16.5, we found that roughly half of the TCKO germ cells expressed SCP3 at E16.5 (Figures 5D and 5E). Although TCKO germ cells in the ovary were positive for SCP3, synaptonemal complex formation in TCKO germ cells was not obvious at this time point. In contrast, control meiotic germ cells exhibited SCP3 loaded onto condensed chromosomes as is typical during the pachytene stage of meiotic prophase I (Figure S5C). RNA-seq from E13.5 PGCs revealed that the TCKO PGCs in the ovary had attenuation expression of the meiotic program, including reduced expression of *Stra8* and *Tex15*, indicative of inefficient differentiation of ovarian PGCs to meiotic germ cells (Figure 5F).

Finally, as some TCKO ovarian germ cells were positive for markers of meiosis, we asked if there was any evidence of oocyte formation in the adult TCKO ovaries at sexual maturity. While TCKO animals fail to thrive post birth, we were able to rear one TCKO female to 6.8 weeks of age. In comparison to a control littermate, we found the TCKO ovary to be much smaller (Figures 5H and S5D). Control ovaries had STELLA-expressing oocytes in primordial follicles as well as secondary and antral follicles. Conversely, one of the two TCKO ovaries lacked oocytes altogether but did contain stroma and granulosa cells, marked by NR2F2 (COUP-TFII) and FOXL2, respectively. The second TCKO ovary contained two activated follicles with atretic-appearing STELLA^+^ oocytes but otherwise lacked a reserve of primordial follicles (Figures 5G and S5E). Although meiotic germ cell development may be indirectly impacted by *Prdm1*-cre knockout of TRIM28 during post-natal development, our data here align with our findings in E16.5 TCKO ovarian germ cells showing improper meiotic progression, suggesting that TRIM28 function during differentiation supports proper meiotic germ cell development.

## Discussion

It is well established that the vast majority of TEs in PGCs are under durable epigenetic repression. Here, we show that TRIM28 serves to maintain durable repression of certain TE subfamilies, most notably ERVs. However, we also show that the chromatin state of certain TE subfamilies in PGCs is highly dynamic, with sex-specific changes in TE accessibility before and during the PGC differentiation window from E10.4–E14.5. The epigenetic regulation of these dynamically accessible TEs does not appear to involve TRIM28. In addition, our TRIM28 PGC conditional knockout study highlights the sex-specific regulation of TEs downstream of TRIM28, particularly at E12.5, as PGCs initiate sex-specific differentiation. Critically, E12.5 is also a major convergence point for PGC epigenetic control. Indeed, PGC-specific deletions of *Dnmt1*, *Prmt5*, *Eed*, *Ezh2*, *Setdb1*, *and Ring1b* lead to either accelerated or stalled sex-specific PGC differentiation at or around E12.5.^35^^,^^44^^,^^45^^,^^46^^,^^47^^,^^72^^,^^73^ In this study, we contribute new knowledge of the sex-specific role of TRIM28 in PGC differentiation.

TRIM28 represses over 3,600 ERVs in the germline of both sexes; however, there are nearly 4,500 additional ERVs that are significantly more accessible in the absence of TRIM28 in testicular germ cells. This sex-specific difference in activity aligns with our observation that TRIM28 becomes less abundant in the ovarian germ line after E12.5. A PGC-specific knockout of *Setdb1*, the downstream effector of TRIM28, also results in weaker TE derepression in ovarian germ cells, indicating that TRIM28/SETDB1 is likely compensated by other mechanisms of TE repression in the female germline, whereas TRIM28/SETDB1 remains a major regulator of ERVs in testicular germ cells after E12.5.^44^ One possible compensatory mechanism of ERV repression in ovarian germ cells is *Ezh2*/H3K27me3, as loss of *Ezh2* (but not *Eed*) results in derepression of young ERVs, similar to some repressed by TRIM28.^45^^,^^72^ In contrast, as *Ezh2* knockout in testicular germ cells does not result in ERV derepression, it is likely that TRIM28 is a major regulator of young ERVs in the testicular germ cells until repression of these ERVs is reinforced by the piRNA pathway.

Proper PGC differentiation entails expression of the competency factor DAZL. When *Dazl* is knocked out, normal repression of pluripotency genes is disrupted and germ cells fail to differentiate correctly.^37^^,^^38^^,^^70^ In TCKO PGCs, we discovered the absence of DAZL from the majority of PGCs at E12.5 with gains in DAZL expression by E13.5, with the ovarian germline showing a more exacerbated failure to induce DAZL. Although a reduction in proliferation-coupled loss of DNA methylation at the *Dazl* locus could explain this result in part, we found differing severities of DAZL protein misregulation when comparing PGCs in the testis and ovary, suggesting that sex-specific regulators contribute to our observed DAZL phenotype.^68^^,^^74^^,^^75^ Failure of TCKO PGCs to appropriately express DAZL did not alter the initial decrease in pluripotency protein expression; however, we did observe that some ovarian PGCs failed to repress SOX2 and NANOG by E14.5. In contrast, testicular PGCs efficiently repressed SOX2 and NANOG but not TFAP2C. Our data show TRIM28 is bound to the gene body of *Dazl*, making it possible that TRIM28 has some undefined direct effect at the *Dazl* locus that remains to be determined. While it is unlikely that TRIM28 is acting as a repressor, it is possible that TRIM28 acts on a regulator of *Dazl* through its E3-SUMO ligase activity, which has been shown to positively regulate other proteins.^76^^,^^77^^,^^78^^,^^79^ Among these is FOXL2, which TRIM28 stabilizes in granulosa cells via E3-SUMO ligation, loss of which results in the non-canonical sex reversal of TRIM28-mutant granulosa cells into Sertoli cells.^80^ Sex reversal of the germ cell differentiation pathway does not occur in TCKO PGCs as ovarian PGCs continue to enter meiosis and testicular germ cells do not upregulate known meiotic genes. It would be informative in future studies to identify targets of TRIM28 SUMOylation in PGCs, and this may lead to the identification of new regulators of PGC differentiation.

While transcriptional misregulation and cell death are often observed following genome-wide derepression of TEs, we found that neither stress nor apoptotic pathways were upregulated in TCKO PGCs. Instead, the response to loss of TRIM28 was stalled proliferation and upregulation of 2C-associated genes, including *Dux* and *Zscan4*, as well as 2C-associated ERVs. Both *in vitro* and *in vivo* evidence links TRIM28 to regulation 2C-associated gene expression via control of *Zscan4* and *Duxf3*, a highly conserved transcription factor that activates during zygotic genome activation in mammals.^81^ In embryonic stem cells (ESCs), TRIM28 scaffolds on LINE1 RNA with *Nucleolin* to direct repression of *Dux*,^63^^,^^64^ and TRIM28 knockout in ESCs induces expression of 2C-associated genes.^82^ Interestingly, the *Dux* locus is accessible in PGCs under wild-type conditions, but *Dux* itself is significantly upregulated in TCKO testicular PGCs at E12.5 and in ovarian PGCs at E13.5. In contrast, *Zscan4c*, which drives 2C-state MERVL expression,^65^ gains accessibility in TCKO PGCs and is also significantly upregulated. We find that in PGCs, TRIM28 likely represses a putative enhancer of *Zscan4* given that we identified TRIM28 binding and H3K9me3 enrichment at this enhancer in PGCs.^66^ In TRIM28 knockout PGCs, germ cell number fails to increase beginning at E12.5, the same time point at which we observed major TE de-repression and emergence of 2C-associated transcripts. While TCKO germ cells in the testis show rapid repression of 2C-associated genes, PGCs in the ovary do not, at least by E13.5. Therefore, with an active *Dux* locus, PGCs appear poised to express 2C-associated genes at E12.5.

In sum, our work demonstrates that TRIM28 is a sex-specific regulator of TE repression that becomes exquisitely important for PGC biology at E12.5, safeguarding PGC differentiation. The loss of TRIM28 in PGCs has only minor consequences before E12.5, with normal numbers of PGCs entering the genital ridge and limited effects on TE or gene expression or TE accessibility. At E12.5, a convergence point of PGC epigenomic regulation and the stage at which *Dazl* should be expressed in all PGCs, the TRIM28 phenotype reveals itself. Facilitated by the normal accessibility of *Dux* in PGCs at E12.5, a TRIM28 loss results in upregulation of TEs and derepression of some 2C-associated genes, which are normally silenced during germ cell differentiation. At the same time, TRIM28 loss compromises the ability of PGCs to rapidly proliferate and dysregulates *Dazl* expression, thus disrupting the normal progression of PGC differentiation and therefore preventing PGCs from undergoing sex-specific differentiation correctly. We use this observation to put forth the hypothesis that TRIM28 serves not only as a direct defense against expression of young TEs in PGCs but also to protect the transcriptome of PGCs during sex-specific differentiation.

### Limitations of the study

This study represents an analysis of TRIM28 function in mouse PGCs with an emphasis on the window of embryonic development between E10.5 and E16.5. The major finding is that TRIM28 has sex-specific roles in TE repression and germ cell differentiation. Limitations of this study include the use of Prdm1-Cre, which is not PGC specific. As a result of this limitation, we had fewer adult samples to study as most TCKO animals failed to thrive after birth. In addition, we were unable to explain how TRIM28 directly or indirectly regulates DAZL expression in PGCs.

## Resource availability

### Lead contact

Reagents may be requested from the lead contact, Amander T. Clark (clarka@ucla.edu).

### Materials availability

No novel reagents were made for this work. The TRIM28 floxed mice, OCT4-IRES-EGFP mice, and Prdm1-Cre (Blimp1-Cre) mice are available from The Jackson Laboratory.

### Data and code availability

• Raw data are available from the corresponding author upon request. Sequencing data generated in this work are available from NCBI GEO under accession: GSE266178.
• No custom code was used in this project.
• Fiji (ImageJ) macros and code for data visualization are accessible at https://github.com/dirussoja/TRIM28_PGCs. Any additional information required to reanalyze the date reported in this paper is available from the lead contact upon request.

## Acknowledgments

We thank the UCLA Broad Stem Cell Research Center (BSCRC) FACS Core, UCLA BSCRC Microscopy Core, UCLA BSCRC Sequencing Core, UCLA Tissue Pathology Core Laboratory, and UCLA Division of Laboratory Animal Medicine. Data were processed using the UCLA IDRE Hoffman2 High Performance Cluster and the UCLA Collaboratory.

This work was funded by the NIH/NICHD R01HD058047 to A.T.C. and F31HD113346 to J.A.D. and a UCLA Molecular Biology Institute Graduate Student Fellowship to J.A.D.

## Author contributions

A.T.C. conceived the project. J.A.D. and Y.T. maintained mouse lines. J.A.D. and Y.T. designed all the experiments. J.A.D., Y.T., A.L.W., A.C.R., and A.J.C. conducted experiments. Y.T. bred and developed the TRIM28 mouse line. J.A.D., X.X., and L.Z. analyzed the data. X.X. and W.L. provided feedback on the bioinformatic code and the manuscript. J.A.D. and A.T.C. drafted the manuscript. We thank A.L.W., X.X., and W.L. for their detailed feedback.

## Declaration of interests

A.T.C. is a member of the ISSCR Board of Directors.

## STAR★Methods

### Key resources table

**Table:** 

REAGENT or RESOURCE	SOURCE	IDENTIFIER
**Antibodies**		

Goat anti-Mouse Vasa Homolog	R and D	AF2030; RRID: AB_2277369
Rabbit anti-KAP1 (TRIM28)	Abcam	ab10484; RRID: AB_297223
Rabbit anti-DAZL	Abcam	ab215718; RRID: AB_2893177
Mouse anti-TFAP2C	Santa Cruz Biotechnology	sc-12762, RRID: AB_667770
Mouse anti-Ki67	BD	556003, RRID: AB_396287
Rabbit anti-Sox2	Abcam	ab97959, RRID: AB_2341193
Rabbit anti-Nanog	Abcam	ab214549, RRID: AB_3668944
Rabbit anti-Piwil2 (MILI)	Abcam	ab36764, RRID: AB_777284
Rabbit anti-Piwil4 (MIWI2)	Thermo Fisher	PA5-31448, RRID: AB_2548922
Rabbit anti-γH2Ax	Cell Signaling Technology	9718, RRID: AB_2118009
Mouse anti-Sycp3	Abcam	ab97672, RRID: AB_10678841
Goat anti-FoxL2	Novus	NB100-1277, RRID: AB_2106188
Mouse anti-Nr2f2 (COUP TF II)	Perseus	PP-H7147-00, RRID: AB_2155627
Rabit anti-Dppa3 (STELLA)	Abcam	ab19878, RRID: AB_2246120

**Critical commercial assays**		

Ovation RNA-Seq System V2	Tecan Genomics	7102
Ovation UltraLow System V2	Tecan Genomics	0344NB-32
Illumina Tagment DNA Enzyme and Buffer Small Kit	Illumina	20034197
Monarch PCR and DNA Clean-UP	New England Biolabs	T1030S
RNeasy Micro	Qiagen	74004
Qiagen MinElute Reaction Cleanup Kit	Qiagen	28204
Agencourt AMPure XP	Beckman Coulter	A63881
NEBNext High-Fidelity 2X PCR Master Mix	New England Biolabs	M0541L
Invitrogen SYBR Green I 10,000X	Invitrogen	S7563
High Sensitivity D1000 Screen Tape	Agilent	5067-5584
KAPA Library Quantification Kits for Next-Generation Sequencing	Kapa Biosystems (Roche)	kk4824
Quibit dsDNA High Sensitivity	Life Technologies	Q32854
Dynabeads Protein A Beads	Invitrogen	10001D
Dynabeads Protein G Beads	Invitrogen	10003D

**Deposited data**		

RNA-seq of PGCs at E11.5, E12.5 and E13.5	This Paper	GEO GSE266176
ATAC-seq of E10.5 and E14.5 PGCs	This Paper	GEO: GSE266172
ChIP-seq of PGCs at E12.5	This Paper	GEO: GSE266173
ATAC-Seq of gonadal soma	Garcia-Moreno et al.^55^	GEO: GSE118755
DNAse-Seq of PGCs	Li et al.^48^	GEO: GSE109770
RNA-seq of mESCs and Zscan4/MERVL+ mESCs	Eckersley-Maslin et al.^61^	GEO: GSE75751
H3K9me3 ChIP-seq of PGCs	Huang et al.^45^	GEO: GSE141180
H3K27ac ChIP-seq of d6 PGCLCs	Kurimoto et al.^49^	GEO: GSE60204
H3K27ac ChIP-seq of E13.5 PGCs	Kawabata et al.^83^	DDBJ: DRA006633

**Experimental models: Organisms/strains**		

Mouse: Strain B6;129S4-*Pouf5f1*^*tm2Jae*^/J	The Jackson Laboratory	RRID: IMSR_JAX:008214
Mouse: Strain B6.Cg-Tg(Prdm1-Cre)^1Masu^/J	The Jackson Laboratory	RRID: IMSR_JAX:008827
Mouse: Strain B6.129S2(SJL)-Trim28^tm1.1Ipc^/J	The Jackson Laboratory	RRID: MSR_JAX:018552
Mouse Strain: CD1	Charles River	RRID: IMSR_CRL:022

**Oligonucleotides**		

Primers are listed in Table S1.	NA	NA

**Software and algorithms**		

STAR 2.7.9a, 2.7.10a	Dobin et al.^84^	RRID: SCR_004463
Subread 2.0.2	Liao et al.^85^	RRID: SCR_012919
Bedtools 2.30.0	Quinlan et al.^86^	RRID: SCR_006646
Samtools 1.15.0	Li et al.^87^	RRID: SCR_002105
R 4.2.1, 4.2.2, 4.3.3	R Core Team^88^	RRID: SCR_001905
DESeq2	Love et al.^89^	RRID: SCR_015687
ggplot2	Wickham^90^	RRID: SCR_014601
DeepTools v3.5.1	Ramirez et al.^91^	RRID: SCR_016366
MACS 3.0.0	Zhang et al.^92^	RRID: SCR_013291
Genrich	John M. Gaspar, https://github.com/jsh58/Genrich	RRID: SCR_025320
SRA Toolkit	US NCBI, https://github.com/ncbi/sra-tools/	RRID: SCR_024350
HOMER	Heinz et al.^93^	RRID: SCR_010881
FIJI	Schindelin et al.^94^	RRID: SCR_002285
Picard tools v2.25.0	Broad Institute, http://broadinstitute.github.io/picard	RRID: SCR_006525
pheatmap	Kolde^95^	RRID: SCR_016418
Smplot2	Min et al.^96^	NA
TrimGalore	Martin^97^*and*https://github.com/FelixKrueger/TrimGalore	RRID: SCR_011841
FastQC	Andrews^98^https://www.bioinformatics.babraham.ac.uk/projects/fastqc/	RRID:SCR_014583
IGV Viewer 2.16.1	Robinson et al.^99^	RRID:SCR_011793
Ggpubr 0.6.0	Kassambara, https://rpkgs.datanovia.com/ggpubr/index.html^101^	RRID:SCR_021139
biomaRt 2.58.2	Durinck et al.^100^	RRID:SCR_019214
Zen Black 2.3 SP1	Zeiss	RRID: SCR_013672
Zen Blue	Zeiss	RRID: SCR_013672
Graphpad Prism	Dotmatics/Graphpad	RRID: SCR_002798
RAD Analysis	Guo^62^	N/A

**Other**		

Zeiss LSM 880	Zeiss	RRID: SCR_020925
Agilent 4200 Tapstation	Agilent	RRID: SCR_018435
Biorad CFX Connect Thermocycler	Biorad	RRID: SCR_017251
Biorad T100 Thermocycler	Biorad	RRID: SCR_021921
Illumina NovaSeq 6000	Illumina	RRID: SCR_016387
Illumina NovaSeq X Plus	Illumina	RRID: SCR_024569
Covaris S2 Sonicator	Covaris	N/A

### Experimental model and study participant details

#### Animal care

All animal experiments were approved by the UCLA Institutional Animal Care and Use Committee (IACUC), also known as the Chancellor’s Animal Research Committee (ARC). All mice were housed in a standard research facility with a 12-hour light/dark cycle and at-will access to standard chow and water. All animals for this study were either bred in UCLA’s on-site facilities or in a Charles River off-site colony facility, both of which adhere to national laboratory animal care guidelines. The institutional approval numbers for this protocol are ARC-2008-070 and ARC-2008-071.

#### Animal models

Trim28^flox/flox^ mice were established in.^101^ Trim28^flox/flox^ (Jackson Laboratory, Cat # 018552) mice on a mixed C57BL/6 129/Sv background. Trim28^flox/flox^ was crossed with Oct4-IRES-eGFP mice^58^ on a C57BL/6 x 129S4/SvJae background (Jackson Laboratory) and backcrossed to get Trim28^flox/flox^;Oct4-eGFP^+/+^ mice. Trim28^flox/flox^;Oct4-eGFP^+/+^ mice were outcrossed to CD1 (Charles River, Cat # 022) with one backcross. Prdm1-cre^24^ mice on a B6CBAF1 background (Jackson Laboratory, 008827) maintained by breeding heterozygous Prdm1-cre males with pre-breeding C57/BL6 females (Jackson Laboratory, Cat# 000664). Prdm1-cre, Trim28^Δ/+^, Oct4-IRES-eGFP^+/-^ males were generated by crossing Prdm1-cre males to Trim28^flox/flox^, Oct4-IRES-eGFP^+/-^ females. Male Prdm1-cre, Trim28^Δ/+^, Oct4-IRES-eGFP^+/-^ were crossed with Trim28^flox/flox^;Oct4-eGFP^+/+^ females to generate Prdm1-cre, Trim28^flox/Δ^ (TCKO) and Trim28^flox/+^ (control) progeny. For all experiments, impregnation was assessed by presence of a vaginal plug, which was considered E0.5. Embryos were harvested for dissection at E10.5, E11.5, E12.5, E13.5, E14.5 and E16.5 in accordance with primary and secondary euthanasia requirements as set out by UCLA IACUC. Embryo sample genotypes were confirmed by PCR for Prdm1-cre, Trim28^flox^ and sex (Table S2).

### Method details

#### Immunofluorescence

Aorta-Gonad-mesonephros of E11.5 and gonads of E11.5, E12.5, E13.5, E16.5, P1 embryos or 48-day adult ovaries were isolated via dissection and fixed in 4% formaldehyde (Thermo Scientific, PI28908 diluted in 1X PBS) overnight at 4C. Samples were then rinsed with 1x PBS and treated for 5 minutes with hematoxylin (Sigma-Aldrich, GHS116) to aid in sectioning. Samples were rinsed once more with 1x PBS and embedded in HistoGel (Epredia, HG-4000) and stored in 70% ethanol. Paraffin embedding and sectioning was either performed by UCLA Tissue Pathology Core Laboratory (TPCL) or by ethanol dehydration followed by xylene and paraffin (Leica Paraplast X-tra, 39603002) perfusion. Samples were prepared as 5 μm sections. Sections were deparaffinized by immersion in xylenes (Fisher Scientific) followed by immersion in 100% (v/v), 95% (v/v), 70% (v/v) and 50% (v/v) ethanol solutions before final immersion in ddH2O and 1x PBS. For antigen retrieval, samples were immersed in a pH 6 solution of 10 mM Sodium Citrate (Sigma Aldrich, S1804) and 0.5% Tween-20 (Fisher Scientific, BP337) at 95C for 40 minutes. Samples were then allowed to cool to room temperature for 20 minutes. Sections were washed with 1x PBS followed by 1x PBS + 0.2% Tween-20 (PBS-T) and permeabilized by washing with 1x PBS + 0.5% Triton-X (MP Biomedicals, 194854) for 20 minutes at room temperature. Samples were washed three times with 1x PBS-T and blocked for 45 minutes at room temperature in 12.5% Normal Donkey Serum in PBS-T (Jackson Immunological Laboratories, 017-000-001). After blocking samples were incubated with primary antibodies (Table S1) in a 2.5% NDS PBS-T solution overnight at 4C in a humid chamber. The following day samples were washed with PBS-T three times for five minutes each. Secondary antibodies conjugated to AlexFluor 488, 594 or 647 (Jackson ImmunoResearch) diluted 1:200 in a 1x PBS-T solution were added to samples which were then allowed to sit overnight at 4C in a humid chamber. For E16.5 gonadal sections the same protocol was followed except samples were blocked for 45 minutes at room temperature using SuperBlock (Thermo Scientific, Ref# 37580), primary antibodies were diluted in Solution 1 (EMD Millipore, KP31812) and secondary antibodies were diluted in Solution 2 (EMD Millipore, KP31855). Samples were then washed 3x with 1x PBS-T and then treated with 1x DAPI (Life Technologies, D1306) in PBS-T for 10 minutes. Samples were mounted in Prolong Gold mounting media (Invitrogen, P36934) and stored to 4-10C prior to imaging.

#### Microscopy

All samples were images on Zeiss LSM880 Axio Observer Z.1 microscopes using Zen Black 2.3 SP1 or on a Zeiss LSM800 Axio Observer using Zen Blue. All sample images were taken as Z-stacks at 40x or 20x magnification. Image processing for publication was performed using FIJI^94^ to perform maximum-intensity projections, adjust brightness for publication, and add scale bars. Adobe Photoshop was used to crop regions of interest and overlay arrows, labels and scalebars prior to publication.

#### Image analysis

Abundance of TRIM28 in PGCs versus soma and yH2Ax abundance was assessed using Imaris 8.3.1 (Bitplane). In brief, spots were assigned and manually verified for soma and PGCs. Soma and PGCs for each technical replicate were averaged and the resulting ratio for each biological replicate reported. cPARP, DAZL, MIWI2, MILI, E16.5 NANOG and SOX2 and E16.5 oocyte γH2AX and SCP3 quantification were done by removing unnecessary z-slices using FIJI (ImageJ, NIH) and counting positive and negative PGCs. Ratios reported are taken from totals across technical replicates for each biological replicate. Ki67, Nanog, Tfap2c, and Sox2 relative intensity quantification were performed using FIJI (ImageJ, NIH) by selecting PGCs and a selection of somatic cells in a supervised manner. The mean gray intensity of individual PGCs was taken, normalized to somatic cells within technical replicates and normalized values plotted. Each PGC quantification is normalized to the somatic average within that technical replicate. Scripts for ImageJ analyses are available upon request. Statistical analysis and graphs generated using Prism 10.2.2.

#### FACS isolation of PGCs

Embryonic trunks (E10.5, E11.5) or embryonic gonads (E12.5, E13.5 or E14.5) were dissected from embryos in 1x PBS and placed in 0.05% Trypsin (Gibco, 25300054) at 37°C/5% CO2 for 5-minute intervals until they were visibly dissociated. Trypsin was quenched using 1x High Glucose DMEM (Gibco, 11965092) + 10% FBS (Gibco, 26140079) and centrifuged at 5,000 rpm for 5 minutes. Samples were washed 1x with 1x DPBS (Gibco, 14190144) + 1% BSA (Sigma-Aldrich, A3311) on ice and centrifuged again at 1200 rpm for 5 minutes. Samples were resuspended in 1x PBS + 1% BSA on ice and passed through a 100 um filter and treated 7AAD (BD Pharmingen, 559925) viability dye. FACS sorting was performed by the UCLA Broad Stem Cell Research Center FACS core using a BD ARIA SORP sorting for GFP+, 7AAD negative cells. For RNA-seq, cells were sorted into Qiagen Buffer RLT (Qiagen, 79216). For ATAC-seq, PGCs were sorted into 1x PBS + 1% BSA.

#### Isolation of PGCs for ChIP-seq

PGCs were isolated from 10 E12.5 time-mated breeding of pure-breeding CD1 mice (Charles River, 022). Entire ovaries or testes from E12.5 embryos, including the mesonephros, were dissected and placed in high glucose DMEM (Gibco, 11965092) + 10% FBS (Gibco, 26140079) on ice. The mesonephros was removed via surgical blades and collected in high glucose DMEM (Gibco, 11965092) + 10% FBS (Gibco, 26140079) with 1x DNase I (Invitrogen, 18047-019) and then washed twice with 1X DPBS (Gibco, 14190144). Resulting ovaries were dissociated in 0.05% Trypsin (Gibco, 25300054) for 10 minutes at 37C. Trypsin was quenched using high glucose DMEM + 10% FBS as above. The resulting suspension was passed through at 70 um cell strainer and then washed using the same media. The pellet was then resuspended in 1X DPBS, 0.1% BSA (Sigma-Aldrich, A3311), 0.5% EDTA (Thermo Fisher Scientific, AM9260G). Suspension was incubated with MACS antibodies against SSEA1 (Miltenyi, 130-094-530) (testicular and ovarian samples) and CD31 (Miltenyi, 130-097-418) (ovarian samples only) at 4°C for 30 min (ovarian samples) or 20 minutes (testicular samples). The resulting cells were centrifuged at 1600 rpm for 5 minutes and resuspended in the same buffer as above and loaded onto an MS column on a MiniMACS magnet. 1 mL of the buffer described above was added to the column and the cells released using the supplied plunger. The resulting suspension was counted and cells fixed as descripted in the ChIP methods.

#### Library preparation for RNA-seq

RNA from PGCs sorted into Qiagen Buffer RLT was extracted using Qiagen RNeasy (Qiagen, 74004) according to the manufacturer’s instructions. cDNA libraries were prepared using Ovation RNA-seq System V2 (Tecan Genomics, 7102) according to manufacturer’s instructions. cDNA libraries were purified using Beckman Coulter Agencourt beads (Beckman Coulter, A63881). Purified cDNA was amplified by SPIA and purified using Qiagen MinElute (Qiagen, 28206) according to manufacturer’s instructions and eluted in 50 uL of low EDTA TE buffer. Libraries were then sonicated to 200 bp fragments using a Covaris S2 (Covaris, Woburn MA) sonicator. Sonicated cDNA was purified using Qiagen MinElute (Qiagen, 28206) according to the manufacturer’s instructions and the cDNA eluted in 10 uL of low EDTA TE buffer. 8uL of eluate was then repaired and indexed for sequencing using Encore Rapid Library System (Tecan Genomics, 0319 and 0320) according to manufacturer’s instructions. Final indexed libraries were purified using Agencourt XP beads (Beckman Coulter, A63881) and the final libraries suspended in low-EDTA TE buffer. Library concentration was assessed using KAPA Library Quantification Kit for Illumina platforms (Roche Sequencing, KK4824) according to the manufacturer’s instructions. Libraries were sequenced on an Ilumina NovaSeq 6000 at 2 x 50 bp (E13.5) or 2x100 bp (E11.5 and E12.5). Sequencing details can be found in Table S4.

#### Library preparation for ATAC-seq

We followed a low-input OMNI-ATAC-seq protocol as in.^102^ For E10.5 samples, all PGCs were collected. For E14.5 samples, we collected only 1500 PGCs per sample, to avoid differences in input quantity between TCKO and control gonads. Immediately following isolation via FACS, sorted PGCs in 1x PBS + 1% BSA were pelleted by centrifugation at 750 rcf for 10 minutes at 4C. Supernatant was aspirated an resuspended in 400 uL of pre-chilled RSB buffer on ice: 10 mM Tris-HCl pH 7.4 (Teknova, 15074), 10 mM NaCl (Invitrogen, AM9760G), 3 mM MgCl_2_ (Invitrogen, AM9530G). Suspension was re-pelleted by centrifugation at 750 RCF for 10 minutes at 4C. Following centrifugation, supernatant was removed and pellet was resuspended in 10 uL of Tn5 Enzyme Mix on ice: 0.33X PBS, 10 mM Tris-HCl ph 7.6, 5 mM MgCl_2_, 5% Dimethyl Formamide (Acros Organics, 327171000), 3mM Tn5 Enzyme (Illumina, 20034197), 0.1% Digitonin (Promega, G9441), 0.1% Tween-20 (Sigma Aldrich, 11332465001), 0.1% NP-40(Sigma Aldrich, 11332473001). Reaction was processed on a thermoshaker at 37C, 1000 rpm for 30 minutes. After reaction, samples were placed on ice and diluted with 10 uL of pre-chilled ice cold water. Fragmented DNA was isolated using NEB Monarch DNA purification system (NEB, T1030S). 100 uL of Monarch DNA Cleanup Binding Buffer was added to each sample and passed through the column by centrifugation for 1 minutes at 13500 rpm. Flow-through was discarded and column washed two times with 200 uL of DNA Wash Buffer followed by centrifugation for 1 minute at 13500 rpm. Samples were eluted in 20 uL of water. Eluate was mixed on ice with 2x NEBNext High-Fidelity 2x PCR mix (NEB, M0541L) and 25 uM of Indexing forward and reverse primers based on Illumina Nextera Indexing Kit (IDT). Resulting reaction was amplified for 5 cycles, then split into two reactions prior to 4 and 8 more cycles of amplification. Amplified DNA was purified using NEB Monarch DNA Purification kit as above using 200 uL of DNA binding buffer and eluting in 21 uL of Low-EDTA EB buffer. Sample quality was assessed using Tapestation 4200 using a D1000 ScreenTap (Agilent Technologies, 5067-5582) and quantified by Quibit HS (Thermo Fisher Scientific, Q32854). Samples were sequenced on an Illumina NovaSeq 6000 (E14.5, SP 2x50 bp) or NovaSeq X (E10.5, 2x100 bp). Sequencing details can be found in Table S4.

#### Chromatin immunoprecipitation (ChIP)

After isolation via MACS as described above, samples were rinsed with 1x PBS (Gibco, 14190144). Cells were pelleted by centrifugation at 1500 rpm for 5 minutes. Supernatant removed. Cells were resuspended in 1% paraformaldehyde (Thermo Scientific Pierce, PI28906) at room temperature rotating for 10 minutes. Fixation was quenched using 0.14 M Glycine (Fisher Scientific, G45212) rotating at room temperature for 10 minutes. Cells were pelleted at 3000 rpm for 5 minutes, supernatant removed and flash frozen in liquid nitrogen and stored at -80C. Fixed PGCs were thawed on ice and resuspended in 10 mM Tris-HCl pH 8 (Invitrogen 15568025), 0.25% Triton X-100 (MP Biomedicals, ICN19485480), 10 mM EDTA (Sigma-Aldrich, T9285), 0.5 mM EGTA (bioWorld, 40520008), Roche cOmplete, 4693116001), 1mM PMSF (Thermo Scientific, 36978) and incubated rotating at room temperature for 15 minutes. Cells were pelleted by centrifugation at 4000 rpm for 5 minutes at 4C. Supernatant was removed and cells resuspended in 10 mM Tris-HCl pH 8.0 (Invitrogen 15568025), 200 mM NaCl (Invitrogen, AM9760G), 10 mM EDTA (Sigma-Aldrich, T9285), 0.5 mM EGTA (bioWorld, 40520008), Roche cOmplete, 4693116001), 1mM PMSF (Thermo Scientific, 36978) for 10 minutes rotating at 4C. Cells were pelleted by centrifugation at 4000 rpm, for 5 minutes at 4C. Supernatant was discarded and pellet resuspended in 650 uL of 10 mM Tris-HCl pH 8.0 (Invitrogen 15568025), 10 mM EDTA (Sigma-Aldrich, T9285), 0.5 mM EGTA (bioWorld, 40520008), Roche cOmplete, 4693116001), 1mM PMSF (Thermo Scientific, 36978). Supernatant was transferred to a 12mm sonication tube (Covaris, 520130) and sonicated using a Covaris S2 with the following program: Intensity = 5, Cycles/burst = 200, duty cycle = 5% with a 4x 30” on, 30” off, 30” on, 30” off periodicity for an effective sonication time of 4 minutes. Sonicated lysate was centrifuged at 14000 rpm for 10 minutes at 4C. 10% of clarified sonicate was kept as an input sample at -80C. Samples to be immunoprecipitated were pre-cleared with Protein A Dynabeads (Invitrogen, 10001D) in a 16.7 mM Tris-HCl pH 8.0 (Invitrogen 15568025), 0.01% SDS (Ambion, AM9820), 1.1% Triton-X 100 (MP Biomedicals, ICN19485480), 1.2 mM EDTA (Sigma-Aldrich, T9285), 167 mM NaCl (Invitrogen, AM9760G) solution for 2 hours rotating at 4C. Supernatant was collected and 4 ug of Abcam ab10484 added. Antibody and supernatant were left to incubate overnight rotating at 4C. Protein A dynabeads were added to mixture as before and incubated rotating at 4C for 2 hours. Supernatant was the removed and discarded and dynabeads washed 2 times with 50 mM HEPES pH 7.9 (Fisher Bioreagents, BP310), 1% Triton-X100 (MP Biomedicals, ICN19485480), 0.1% Deoxycholate, 1mM EDTA (Sigma-Aldrich, T9285), 140 mM NaCl (Invitrogen, AM9760G) for 4 minutes rotating at 4C. Beads were then washed two times with 2x with Tris-EDTA for 4 minutes rotating at 4C. Supernatant was discarded and beads resuspended in 50 mM Tris-HCl pH 8 (Invitrogen 15568025), 1mM EDTA (Sigma-Aldrich, T9285), 1% SDS (Ambion, AM9820) for elution. Elution was performed on a thermoshaker at 65C shaking at 1400 rpm for 10 minutes. Supernatant was collected and the elution procedure repeated on beads. First and second elution were pooled. Input fractions and eluted chromatin were incubated overnight at 65C. Samples were then treated with 15 ug of RNAseA (Invitrogen, 12091021) for 30 minutes at 37C. Samples were then treated with 100 ug of Proteinase K for 2 hours at 56C. DNA was then purified using Quiagen MinElute (Qiagen, 28206) according to instructions with a final elution of 12 uL.

#### ChIP-seq library preparation

ChIP-seq libraries were prepared using the Ovation UltraLow System V2 (Tecan Genomics, 0344) according to manufacturer’s instructions. For each library, the number of amplification cycles was based off of quantification by Quibit dsHS (Thermo Fisher Scientific, Q32854). Final amplified and indexed libraries were purified using Agencourt XP beads (Beckman Coulter, A63881) and library quantity determined using Quibit HS assuming an average fragment length of 250 bp. Libraries were sequenced on an Illumina Novaseq 6000 at 2x100 bp. Sequencing details can be found in Table S4.

#### Bioinformatic analysis

##### Data retrieval, reference genome and repeatmasker

Mouse reference genome GRCm39 and the corresponding ENSEMBL gene annotation file GRCm39.109 were downloaded from Ensembl and utilized for all genomics analyses. TE annotation file for GRCm39 from repeatmasker (http://repeatmasker.org/) were used for TE related analyses. To retrieve data from US NCBI SRA, we used SRA Tools v2.10.9 (US NCBI). For the online datasets used in this study, refer to Table S3.

##### RNA-seq

Quality control and read quantification were adopted and optimized from a previous study.^13^ Specifically, we used FastQC v0.11.9^98^ to perform quality control on raw RNA-seq data. The alignment was performed with STAR 2.7.10a^84^ with the optimized setting (--outFilterMultimapNmax 1000, --outSAMmultNmax 1, --outFilterMismatchNmax 3, and --alignIntronMax 1). We sorted and indexed the aligned bam output using SAMtools 1.15^87^ with the default setting. The read quantification was performed separately for genes and transposable elements. We used the featureCounts function from Subread 2.0.2^85^ to process the BAM outputs from the previous step. For TE quantification, we included multimapped reads and quantified the individual duplicates (transcript_id), as well as individual subfamilies (gene_id).

The raw read counts were input into and processed by R 4.2.2.^88^ Genes and TEs with no reads across all samples were removed, and RPKM values were calculated. To identify DETEs and DEGs, we filtered out TEs or genes with RPKM mean less than 1 in both treatment and control groups. Significant TEs and genes were defined with two log2-transformed fold-change thresholds (1.5 and 2) and FDR < 0.05 using DESeq 2.^89^ Both TE and gene expression counts were normalized using the rlogtranformation function and were compared both within each sex and between sexes from all time-points.

To compare TRIM28 KO PGCs and 2C Like Cells from,^61^ reads were aligned as above. Differential gene expression between double-negative E14 mESC cells and Zscan4+/MERVL+ mESCs as identified in the study was done using DESeq2.^89^ ENSMBL IDs were converted to gene names using biomaRt 2.58.2^100^ using ENSEMBL database version 109 (mm39). Gene list from^61^ was used to compare correlation, with any genes lacking a calculable p-adjust value or log2FoldChange value discarded. Correlation was plotted using ggplot2^90^ and correlation calculated using Smplot2^96^ on R 4.3.3. For PCA analysis, E11.5, E12.5 and E13.5 control and mutant PGCs of each sex were normalized with mESC and 2CLC data from^61^ using DESeq2 in R 4.3.3 and PCA generated from the top 750 genes using rld normalized reads in runPCA. For 2C-associated genes, read counts from both sexes, control and TCKO PGCs at E11.5, E12.5 and E13.5 were normalized together using DESeq2 and rld normalized values plotted. Significance is from sex-and-time specific comparisons between control and TCKO PGCs using DESeq2 calculated Log2FoldChange and padj.

##### ChIP-seq

ChIP-seq of TRIM28 were preprocessed using TrimGalore v0.6.10^97^^,^^98^^,^^103^ with the arguments --stringency 3 --length 20 --paired --nextseq 20. Alignment was performed using STAR 2.7.9a with options --outFilterMultimapNmax 1000 --outFilterMismatchNmax 3 --alignIntronMax 1 --outSAMmultNmax 1. For TRIM28 ChIP-seq we invoked --alignEndsType EndToEnd. Deduplication was done using Picard Tools MarkDuplicates 2.25.0.^104^ The aligned BAM files were then processed by SAMtools v1.15^87^ to sort by indexes. For H3K27ac ChIP-seq in d6 PGCLCs, reads were converted from color space to base space using SRA Tools fastq-dump -B.

Bigwig files were generated using Deeptools v3.5.1^91^ bamCoverage with options –bs10 and -e and were normalized by RPKM or normalized read counts. Input-subtracted RPKM bigwig files were generated using bamCompare with --scaleFactorsMethod None --normalizeUsing RPKM -e --operation subtract. Tracks were visualized as heatmaps using the computeMatrix function using the center of the region as the reference point and a window size of 3,000, 5,000 or 10,000 bp up and down-stream invoking --missingDataAsZero. Heatmaps were then generated using plotHeatmap from DeepTools v3.5.1. Profiles were generated using plotProfiles from deepTools v3.5.1. Gene tracks produced using IGV 2.16.1.^99^

ChIP-seq peaks were defined using the callpeak function from MACS v3.0.0^92^ with an FDR threshold of 0.05 and an effective genome size compiled for GRCm39. Peaks were called individually for each biological replicate, combined, coordinate sorted using bedtools sort and overlapping peaks removed by bedtools merge -I -d 20 to produce a final list.

To calculate RPKM of H3K9me3 over TRIM28-derepressed ERVs we calculated RPKM from input-subtracted RPKM bigwig files using DeepTools multiBigwigSummary. For each sex, sex-specific and shared TCKO regions were combined. Graphs of H3K9me3 enrichment were plotted using ggplot2^90^ and ggpubr^105^ using R 4.3.3.

##### ATAC-seq analysis

Adapter trimming on ATAC-seq libraries was performed using TrimGalore v0.6.10^97^^,^^98^^,^^103^ with the parameters --clip_R1 18 --stringency 3 --length 20 --paired --nextseq 20. Trimmed reads were aligned with STAR 2.7.9a^84^ with options --outFilterMultimapNmax 1000 --outFilterMismatchNmax 3 --alignIntronMax 1 --outSAMmultNmax 1 --outFilterMatchNminOverLread 0.2. PCR duplicates were removed using Picard Tools MarkDuplicates 2.25.0.^104^ Biological replicates passing QC were merged using Samtools merge 1.15.^87^ Bigwig files were generated using Deeptools v3.5.1.^91^ Gene tracks produced using IGV 2.16.1.^99^

For analysis of open to closed or closed to open chromatin, peaks of both sexes in control-only conditions were called using Genrich 0.6.0^106^ and merged as above. We then calculated the RPKM of these peaks across all samples using multiBigWigSummary. We set an RPKM threshold of < 15 RPKM for “closed” and > 15 RPKM for “open” chromatin. We then binned, for each sex, peaks which were open at both time points, closed, or transitioned from closed to open or open to closed. Resulting peak files were stored as BED for downstream analysis and visualization of open to closed and closed to open peak classes was performed using computeMatrix –missingDataAsZero followed by plotHeatmap from deepTools v3.5.1. For gonadal soma ATAC-seq, we used the same approached but used RPKM < 10 as a cutoff for closed and RPKM > 10 as a cutoff for open chromatin. Subfamily tally was performed by dividing the number of unique integrants of a subfamily by the total number of integrants in the subfamily, resulting in the ratio of differentially accessible TEs within each subfamily.

For TRIM28-specific ERV derepression analysis (Figure 2), peaks were called using Genrich 0.6.1^106^ with individual BAMs for biological replicates, with options -j -q 0.05. Peak files for all conditions were merged together and duplicate or bookended peaks removed with bedtools merge -I -d 10.^86^ RPKM of merged peak files were called using multiBigwigSummary. Peaks were classified based on higher RPKM value than all other conditions (across genotype, time and sex) and > 2 fold higher RPKM values than all other conditions in R 4.2.1.^88^ Resulting tables were exported and visualization performed using computeMatrix with –missingDataAsZero followed by plotHeatmap.

For RAD analysis, we used DEG lists from previous 2C analysis and performed lift-over analysis on XY- and XX-ectopic ERVs (including XY/XX shared ERVs) and used the platform in^62^ to generate RAD figures.

##### HOMER motif analysis

Motif analysis was performed using BED files of TEs which transitioned from open to closed or closed to open in our PGC or gonadal soma ATAC-seq analysis. Motif analysis was performed using HOMER v4.11.1.^93^ Motifs were called using findMotifsGenome.pl with --mset vertebrates against the mm39 genome. Motifs from knownMotifs were plotted using R 4.3.3 using pheatmap^95^ with p-values from HOMER transformed to -log10(pvalue) in R 4.3.3.

##### DNAse-analysis

FASTQ files were aligned using STAR v2.7.9a^84^ with the parameters --outFilterMultimapNmax 1000 --outFilterMismatchNmax 3 --alignIntronMax 1 --outSAMmultNmax 1. PCR duplicates were removed using Picard Tools MarkDuplicates 2.25.0.^104^ Biological replicates passing QC were merged using Samtools merge 1.15.^87^ Bigwig files were generated using Deeptools v3.5.1.^91^ Peaks were called using MACS3^92^ with parameters -f BAM -g 2654621783 -q 0.01. bigWig files were generated using deepTools v3.5.1 bamcoverage. Gene tracks produced using IGV 2.16.1.^99^

### Quantification and statistical analysis

Statistical tests were performed using R (v4.2.1, v4.3.3) and Graphpad Prism (v 10.2.2-3). p < 0.05 used for all t-test and Welch’s t-test as the cutoff for significance. For RNA-seq, significance was determined by DESeq2 with |Log2FC| > 2 and FDR (p.adj) < 0.05. Significance testing for ATAC-seq and ChIP-seq peak-calling determined by peak-calling program and are detailed in their respective methods. All graphs show individual Ns, and graph details are provided in each figure legend. Source data are provided as Supplemental Tables.

## Supplemental information

Document S1. Figures S1–S5 and Tables S1–S3

Table S4. Read statistics table of read alignment statistics for all datasets used in this study, including those that were obtained through public repositories

Table S5. List of transposable element loci, which change accessibility during PGC differentiation, related to Figure 1A Contains the loci of peaks and overlapping TEs identified in the analysis in 1A and used for Figure S2A.

Table S6. List of ERVs identified as being control XY, XX- or TCKO XX, XY-specific or shared, related to Figures 2C–2E and S2E Lists identified peaks and their associated overlapping ERV across all permutations.

Table S7. DE genes and TEs from RNA-seq at E11.5, E12.5, E13.5, related to Figures 2F, S2F, S2G, 3A–3C, S3A, and S3B List of all DETEs and DE Genes compared time-matched and sex-matched control vs. TCKO conditions.

Table S8. 2C-DE gene analysis, related to Figures 3B and S3B Ensembl ID, Gene Name, and Log2 Fold Change for TCKO/Control PGCs and Zscan4/MERVL+ mESCs/ Zscan4/MERVL-mESCs.
