# Supplementary material for: Sex-specific expression and function of TRIM28 during mouse primordial germ differentiation
Source: iScience. 2025 Sep 1;28(10):113474. doi: 10.1016/j.isci.2025.113474 (PMC12475620; doi:10.1016/j.isci.2025.113474)
Supplement: Document S1. Figures S1–S5 and Tables S1–S3 [file mmc1.pdf]

iScience, Volume *28*

Supplemental information

Sex-specific expression and function of TRIM28

during mouse primordial germ differentiation

Jonathan A. DiRusso, Lingyu Zhan, Yu Tao, Allison L. Wang, Xinyu Xiang, Alexander C. Robbins, Azra J. Cruz, Wanlu Liu, and Amander T. Clark

Supplemental Figures:

Figure S1: Dynamic Accessibility of Transposable Elements, Related to Figure 1

Figure S2: Direct and Indirect Targets of TRIM28 in Primordial Germ Cells, Related to Figure 2

Figure S3: TRIM28 is Required for Proper Regulation of the PGC Transcriptome, Related to Figure 3

Figure S4: PGCs Inefficiently Enter Differentiation in TRIM28 KO PGCs, Related to Figure 4

Figure S5: TRIM28 is Required for Gametogenesis in both Sexes, Related to Figure 5

Supplemental Tables:

Table S1: Antibodies Used in this Study and Their Dilutions, Related to STAR methods

Table S2: Genotyping Oligos Used in this Study, Related to STAR methods

Table S3: Publicly Available Datasets Used in this Study, Related to STAR methods

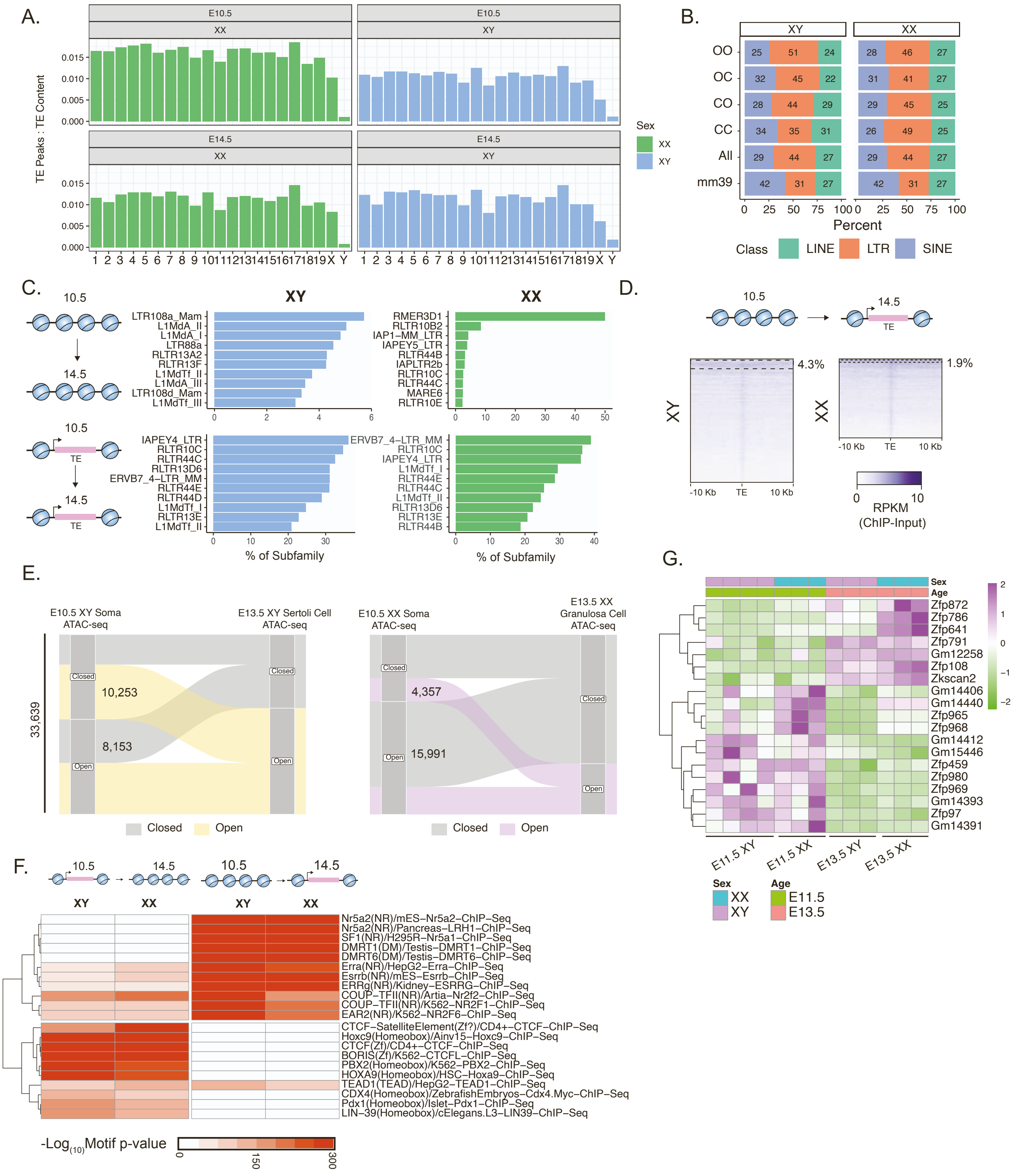

Figure S1: Dynamic Accessibility of Transposable Elements, Related to Figure 1

A) Proportion of peaks called overlapping with TEs relative to the TE load (ERV, LINE and SINE) of each chromosome at E10.5 and E14.5 Color denotes sex. B) Percentage of peaks overlapping with TEs by category (Open to Closed, Closed to Open, Closed to Close and Open to Open) as well as all peaks overlapping with TEs (aggregate) and all TEs in the mm39 repeatmasker database. C) Plots showing the most represented TEs which are constitutively closed (top) or open (bottom) in XY and XX PGCs. Percentage refers to percentage of all elements in a subfamily represented. D) H3K27ac enrichment in E13.5 gonads (XY or XX) in peaks that transition from closed to open. Dotted line and percentage refer to the overlap between H3K27ac peaks and identified TEs. E) Alluvial plots showing the change in TE accessibility in E10.5 and E13.5 soma from XY and XX gonads. F) Motif analysis via HOMER showing enrichment of TE-embedded motifs in TEs which transition from open to closed (left) and close to open (right) in gonadal soma between E10.5 and E13.5. G) Heatmap showing expression of KRAB-ZFPs which are significantly different between E11.5 and E13.5 in both XY and XX germ cells.

<u>S2:</u>

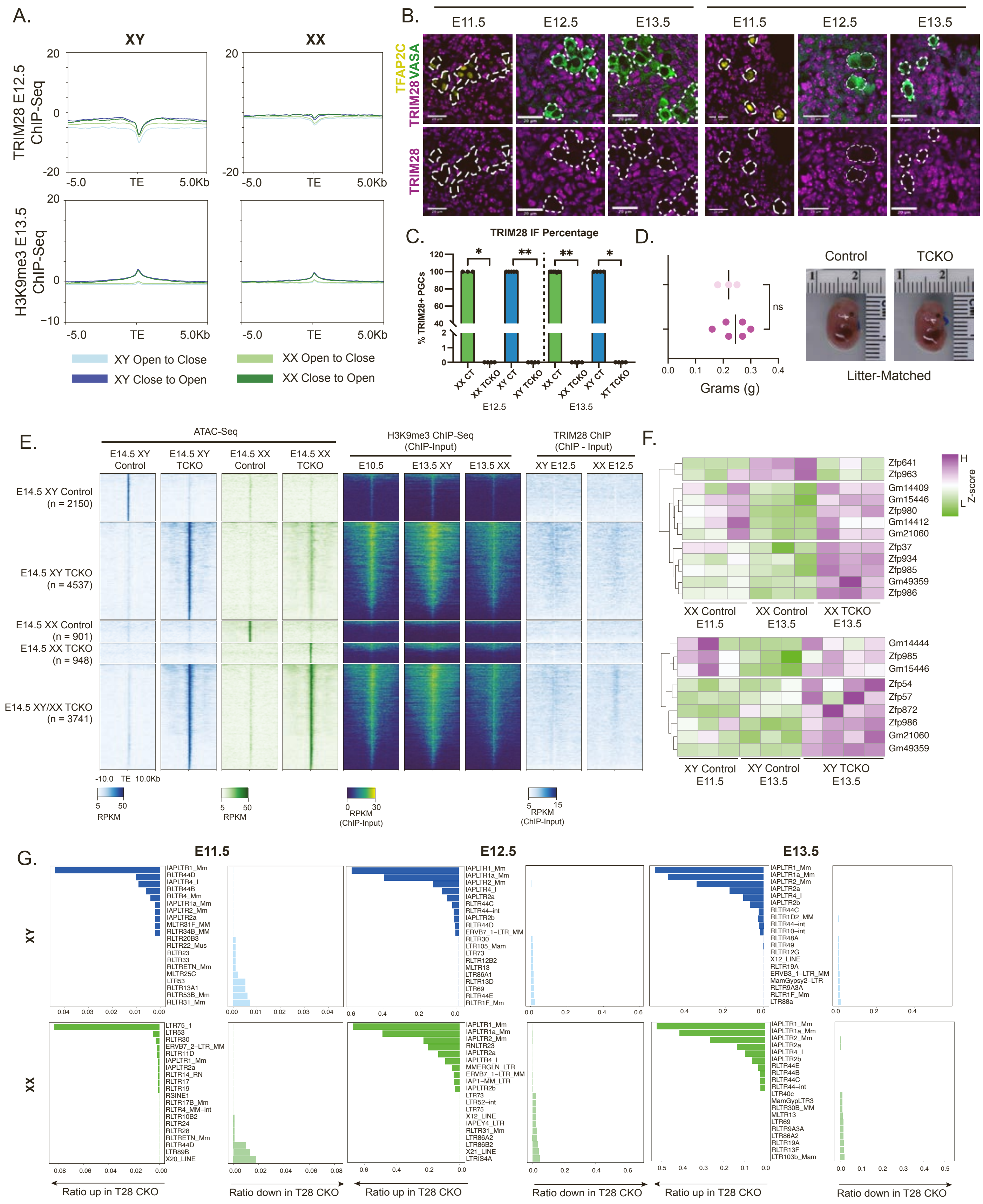

A.
XY
XX
TRIM28 E12.5 ChIP-Seq
H3K9me3 E13.5 ChIP-Seq
-5.0
TE
5.0Kb
XY Open to Close
XY Close to Open
XX Open to Close
XX Close to Open
B.
XY
XX
E11.5
E12.5
E13.5
TFAP2C VASA TRIM28
TRIM28
C.
TRIM28 IF Percentage
% TRIM28+ PGCs
XX CT
XX TCKO
XY CT
XY TCKO
XT TCKO
E12.5
E13.5
D.
ns
Grams (g)
Control
TCKO
Litter-Matched
E.
ATAC-Seq
E14.5 XY Control
E14.5 XY TCKO
E14.5 XX Control
E14.5 XX TCKO
H3K9me3 ChIP-Seq (ChIP-Input)
E10.5
E13.5 XY
E13.5 XX
TRIM28 ChIP (ChIP - Input)
XY E12.5
XX E12.5
E14.5 XY Control (n = 2150)
E14.5 XY TCKO (n = 4537)
E14.5 XX Control (n = 901)
E14.5 XX TCKO (n = 948)
E14.5 XY/XX TCKO (n = 3741)
-10.0 TE 10.0Kb
RPKM
RPKM (ChIP-Input)
F.
Zfp641
Zfp963
Gm14409
Gm15446
Zfp980
Gm14412
Gm21060
Zfp37
Zfp934
Zfp985
Gm49359
Zfp986
XX Control E11.5
XX Control E13.5
XX TCKO E13.5
Gm14444
Zfp985
Gm15446
Zfp54
Zfp57
Zfp872
Zfp986
Gm21060
Gm49359
XY Control E11.5
XY Control E13.5
XY TCKO E13.5
H
L
Z-score
G.
E11.5
E12.5
E13.5
XY
XX
Ratio up in T28 CKO
Ratio down in T28 CKO

Figure S2: Direct and Indirect Targets of TRIM28 in Primordial Germ Cells, Related to Figure 2

A) TRIM28 ChIP-seq enrichment at E12.5 (Top) and H3K9me3 enrichment at E13.5 (bottom) TEs which transition from open to closed or closed to open. In testicular and ovarian PGCs. Y axis is input-subtracted normalized read count. B) Representative IF showing TRIM28 abundance at E11.5, E12.5 and E13.5 via IF. Scale bars represent 20 μm. C) Quantification of TRIM28 knockout. Error bars represent SD. Significance by Kolmogorov-Smirnov test. * $p < 0.05$. D) Mass (left) and morphology (right) of E14.5 embryos from Control and TCKO-PGC harboring embryos. Embryos shown are litter-matched. E) Heatmap showing read density over ATAC-peaks and read density of H3K9me3 and TRIM28 ChIP-seq. ChIP units are input-subtracted RPKM. F) DEG analysis of KRAB-ZFPs significantly misregulated in XY (top) and XX (bottom) PGCs at E13.5. Scale is z-score of normalized (rlog, DESeq2) expression. G) Quantification showing subfamily-level derepression of TE RNAs at E11.5, E12.5 and E13.5. Derepressed TEs are represented on the left, repressed TEs on the right. X-axis represents ratio of elements detected to all elements in subfamily.

S3:

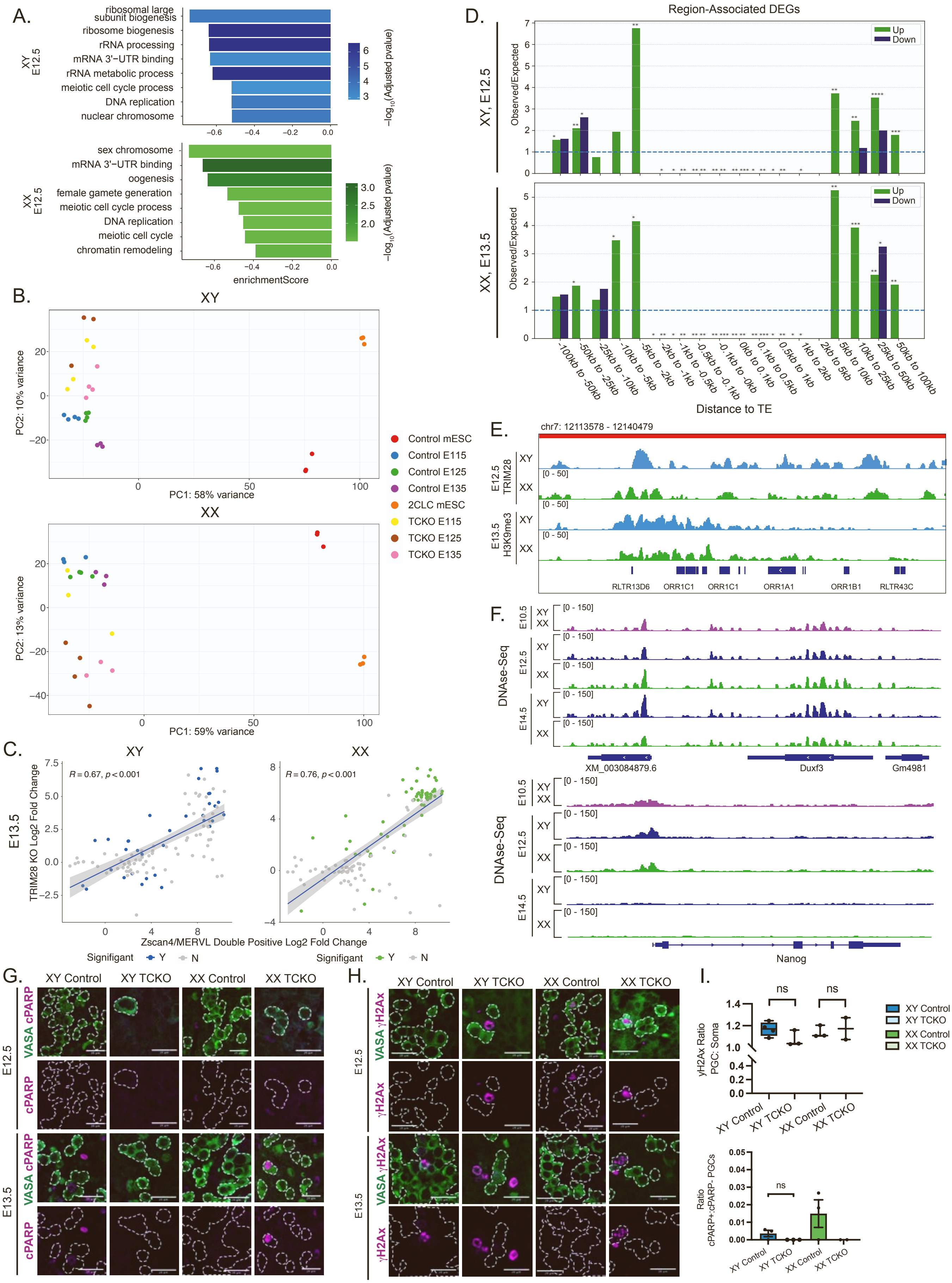

A.
XY E12.5
ribosomal large subunit biogenesis
ribosome biogenesis
rRNA processing
mRNA 3'−UTR binding
rRNA metabolic process
meiotic cell cycle process
DNA replication
nuclear chromosome
−log10(Adjusted pvalue)
XX E12.5
sex chromosome
mRNA 3'−UTR binding
oogenesis
female gamete generation
meiotic cell cycle process
DNA replication
meiotic cell cycle
chromatin remodeling
enrichmentScore
B.
XY
PC2: 10% variance
PC1: 58% variance
XX
PC2: 13% variance
PC1: 59% variance
Control mESC
Control E115
Control E125
Control E135
2CLC mESC
TCKO E115
TCKO E125
TCKO E135
C.
XY
XX
E13.5
TRIM28 KO Log2 Fold Change
R = 0.67, p < 0.001
R = 0.76, p < 0.001
Zscan4/MERVL Double Positive Log2 Fold Change
Signifigant Y N
D.
Region-Associated DEGs
XY, E12.5
XX, E13.5
Observed/Expected
Up
Down
Distance to TE
-100kb to -50kb
-50kb to -25kb
-25kb to -10kb
-10kb to -5kb
-5kb to -2kb
-2kb to -1kb
-1kb to -0.5kb
-0.5kb to -0.1kb
-0.1kb to -0kb
0kb to 0.1kb
0.1kb to 0.5kb
0.5kb to 1kb
1kb to 2kb
2kb to 5kb
5kb to 10kb
10kb to 25kb
25kb to 50kb
50kb to 100kb
E.
chr7: 12113578 - 12140479
E12.5 TRIM28
E13.5 H3K9me3
XY
XX
[0 - 50]
RLTR13D6
ORR1C1
ORR1C1
ORR1A1
ORR1B1
RLTR43C
F.
DNAse-Seq
E10.5
E12.5
E14.5
XY
XX
[0 - 150]
XM_003084879.6
Duxf3
Gm4981
Nanog
G.
XY Control
XY TCKO
XX Control
XX TCKO
E12.5
E13.5
VASA cPARP
cPARP
H.
XY Control
XY TCKO
XX Control
XX TCKO
E12.5
E13.5
VASA γH2Ax
γH2Ax
I.
ns
ns
γH2Ax Ratio PGC: Soma
XY Control
XY TCKO
XX Control
XX TCKO
Ratio cPARP+:cPARP- PGCs
ns

Figure S3: TRIM28 is Required for Proper Regulation of the PGC Transcriptome, Related to Figure 3

A) GO-enriched GSEA analysis of E12.5 XY and E12.5 XX TCKO vs Control PGCs. B) PCA analysis of XY (top) and XX (bottom) PCA cluster of TCKO and control PGCs at E11.5, E12.5 and E13.5 with mESC and 2CLC from Eckersley-Maslin et al, 2016. C) Correlation plots with TCKO PGCs and 2CLCs at E13.5 as in Fig. 3. Correlation is Pearson correlation calculated using smplot2. D) RAD analysis showing relationship between differentially expressed 2C-associated genes and ectopically accessible ERVs at E14.5 in XY and XX TCKO PGCs. E) Gene tracks showing E12.5 TRIM28 ChIP (top) and E13.5 H3K9me3-ChIP (bottom) at a putative Zscan4 enhancer identified in Le, R. et al., (2021) Cell Stem Cell. Tracks are readcount-normalized input-subtracted (deepTools). F) Gene tracks of DNAse-seq from Li, J.et al. (2018) Cell Res. at E10.5, E12.5 and E13.5 from XY and XX PGCs at the *Dux* locus (top). Gene tracks as above for the *Nanog* locus (bottom). G) Representative images of cPARP (magenta) in PGCs (green) at E12.5 and E13.5. Scale bars represent 20 μm. H) Representative images of yH2Ax (magenta) in E12.5 and E13.5 PGCs. Scale bars represent 20 μm. I) Quantification of yH2Ax (top) and cPARP (bottom) ratios at E12.5. Significance testing by Welch's t-test for samples with $n \geq 3$. Error bars are SEM. Each dot represents a biological replicate. Boxplot line represents the mean, box bounds are the first and third quartiles. Whiskers represent maximum and minimum values within 1.5x the first (minimum) or third (maximum) quartile.

S4:

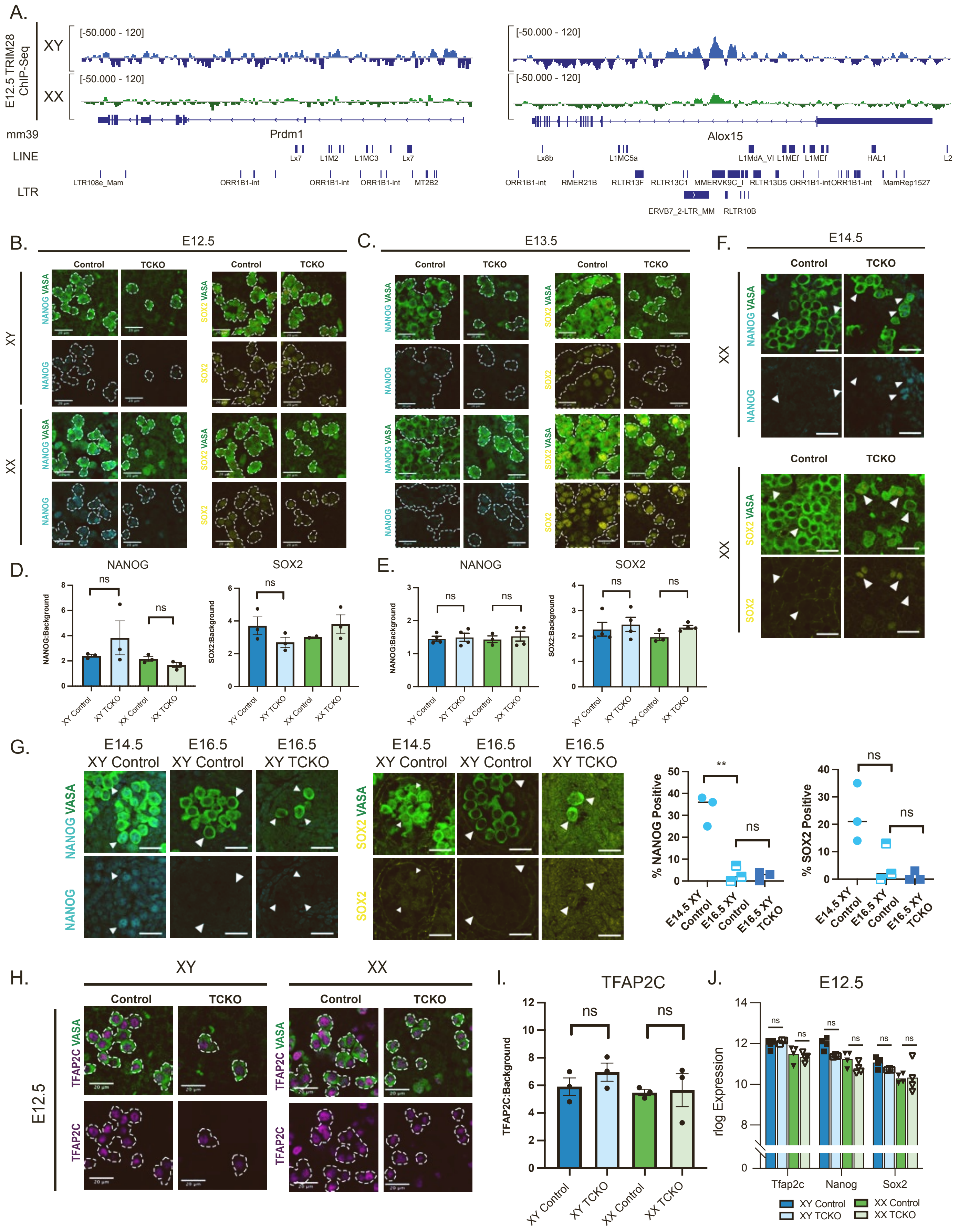
A.
E12.5 TRIM28 ChIP-Seq
XY
XX
[-50.000 - 120]
mm39
Prdm1
Alox15
LINE
LTR
Lx7
L1M2
L1MC3
Lx8b
L1MC5a
L1MdA_VI
L1MEf
HAL1
L2
LTR108e_Mam
ORR1B1-int
MT2B2
RMER21B
RLTR13F
RLTR13C1
MMERVK9C_I
RLTR13D5
MamRep1527
ERVB7_2-LTR_MM
RLTR10B
B.
E12.5
C.
E13.5
F.
E14.5
Control
TCKO
NANOG VASA
NANOG
SOX2 VASA
SOX2
D.
E.
NANOG
SOX2
NANOG:Background
SOX2:Background
ns
XY Control
XY TCKO
XX Control
XX TCKO
G.
E14.5 XY Control
E16.5 XY Control
E16.5 XY TCKO
% NANOG Positive
% SOX2 Positive
**
H.
E12.5
TFAP2C VASA
TFAP2C
I.
TFAP2C
TFAP2C:Background
J.
E12.5
rlog Expression
Tfap2c
Nanog
Sox2

Figure S4: PGCs Inefficiently Enter Differentiation in TRIM28 KO PGCs, Related to Figure 4

A) Gene tracks showing enrichment of TRIM28 at E12.5 (Input-subtracted, read-count normalized) at *Prdm1* (left) and *Alox15* (right). Repeatmasker tracks for ERV, LINE and SINE elements shown below. *Alox15* has intronic ERVs which are repressed by TRIM28. B) Representative images showing expression of NANOG (left, cyan), SOX2 (right, yellow) and VASA (green) in PGCs at E12.5. Scale bars represent 20μm. Note, the same testicular sample slide used for NANOG/VASA (left) was re-used in H with a different stain. C) As in B, but at E13.5. D) Quantification of NANOG and SOX2 relative abundance at E12.5. Y-axis represents ratio of NANOG or SOX2 in PGCs relative to soma. Significance testing by 2-sided unpaired Welch's t-test. Individual points are one biological replicate. Error bars show SEM. E) Same as in D) but for E13.5. F) Representative images of E14.5 ovaries from control (left) and TCKO (right) gonads. Arrows indicated PGCs for orientation. Scale bars represent 20 μm. N=2 each condition. G) (Right) Representative images of testes at E14.5 and E16.5 in control (E14.5 and E16.5) and TCKO (E16.5) conditions. N=3 for each condition. Scale bars represent 20 μm. (Left) Quantification of percentage of NANOG or SOX2+ PGCs from the listed condition. N =3 replicates each. Line represents mean. Each point is 1 biological replicate. Stats are student's t-test. H) Representative images of TFAP2C (magenta) and VASA (green) in testicular and ovarian PGCs at E12.5. Scale bars represent 20 μm. N=3 for all conditions. The same testicular sample slide was re-used in B. I) Quantification of TFAP2C abundance in PGCs at E12.5. Significance testing by unpaired 2-sided Welch's t-test. Individual points are average of all PGCs in biological replicate. Error bars show SEM. J) Normalized expression of *Tfapc2c*, *Nanog* and *Sox2.* Statistical testing by DESeq2. Normalized expression in rlog. For t-tests, ns = $p > 0.05$, * = $p<0.05$, ** = $p<0.01$, *** = $p<0.001$, ****=$p<0.0001$

S5:

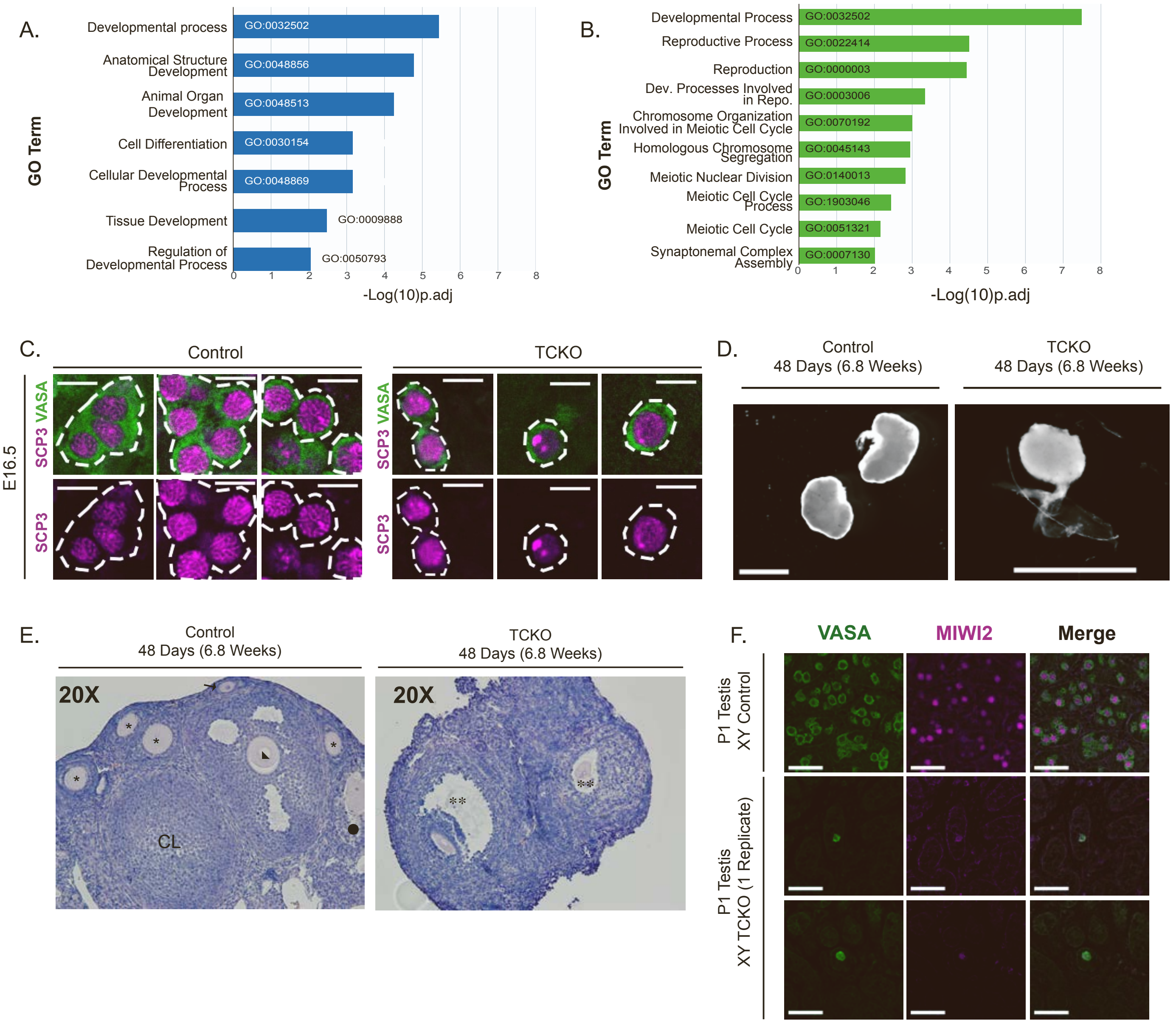
A.
GO Term
Developmental process
Anatomical Structure Development
Animal Organ Development
Cell Differentiation
Cellular Developmental Process
Tissue Development
Regulation of Developmental Process
GO:0032502
GO:0048856
GO:0048513
GO:0030154
GO:0048869
GO:0009888
GO:0050793
-Log(10)p.adj
B.
GO Term
Developmental Process
Reproductive Process
Reproduction
Dev. Processes Involved in Repo.
Chromosome Organization Involved in Meiotic Cell Cycle
Homologous Chromosome Segregation
Meiotic Nuclear Division
Meiotic Cell Cycle Process
Meiotic Cell Cycle
Synaptonemal Complex Assembly
GO:0032502
GO:0022414
GO:0000003
GO:0003006
GO:0070192
GO:0045143
GO:0140013
GO:1903046
GO:0051321
GO:0007130
-Log(10)p.adj
C.
Control
TCKO
E16.5
SCP3 VASA
SCP3
D.
Control 48 Days (6.8 Weeks)
TCKO 48 Days (6.8 Weeks)
E.
Control 48 Days (6.8 Weeks)
TCKO 48 Days (6.8 Weeks)
20X
CL
F.
VASA
MIWI2
Merge
P1 Testis XY Control
P1 Testis XY TCKO (1 Replicate)

Figure S5: TRIM28 is Required for Gametogenesis in both Sexes, Related to Figure 5

A and B) GO analysis of E13.5 testicular PGC differential gene expression using downregulated genes in A) testicular germ cells and B) ovarian germ cells. GO number inset. C) Close-up view of E16.5 meiotic germ cells showing Sycp3 localization at E16.5 in control (left) and TCKO (right) PGCs. Each panel represents 1 biological replicate (n=3 each). Scale bars represent 10 μm. D) Images of ovaries isolated from n=1 48 day-old (~7 weeks) showing whole ovary morphology. Scalebar represents 1500 μm (TCKO ovary is taken at a higher magnification). E) H & E stain of control and TCKO ovary at 48 days. Control ovary shows primary follicle (arrow), secondary follicles (asterisks), antral follicle (arrowhead) and an activated follicle (circle). TCKO ovary shows two malformed follicles, one which appears antral-like (left, two asterisks) and one which appears activated (right, one asterisk). F) IF images from P1 male testes from control (n = 3, top) and TCKO (n=1, bottom), showing reduced PGC number and improperly localized MIWI2 expression. Scale bars represent 50 μm.

Table S1: Antibodies used in this study with their dilutions, Related to STAR Methods

Antibody	Manufacturer	Catalog #	Concentration	Block	Primary Incubation	Secondary Incubation
MVH (VASA)	R and D	AF2030	1:100	12.5% NDS	2.5% NDS	0% NDS
TRIM28	Abcam	ab10484	1:200	12.5% NDS	2.5% NDS	0% NDS
DAZL	Abcam	ab215718	1:200	12.5% NDS	2.5% NDS	0% NDS
SCP3	Abcam	ab97672	1:200	12.5% NDS	2.5% NDS	0% NDS
NANOG	Abcam	ab214549	1:200	12.5% NDS	2.5% NDS	0% NDS
SOX2	Abcam	ab97959	1:200	12.5% NDS	2.5% NDS	0% NDS
TFAP2C	Santa Cruz	Sc-12762	1:200	12.5% NDS	2.5% NDS	0% NDS
PIWIL2 (MILI)	Abcam	ab36764	1:100	SuperBlock	Solution 1	Solution 2
PIWIL4 (MIWI2)	Thermo Scientific	PA5-31448	1:100	SuperBlock	Solution 1	Solution 2
cPARP	Cell Signaling Technology	9544	1:200	12.5% NDS	2.5% NDS	0% NDS
yH2Ax	Cell Signaling Technology	9718	1:200	12.5% NDS	2.5% NDS	0% NDS
Ki67	BD Pharmagen	556003	1:100	12.5% NDS	2.5% NDS	0% NDS
FOXL2	Novus	NB100-1277	1:200	12.5% NDS	2.5% NDS	0% NDS
NR2F2	Perseus	PP-H7147-00	1:200	12.5% NDS	2.5% NDS	0% NDS
DPPA3	Abcam	ab19878	1:500	12.5% NDS	2.5% NDS	0% NDS

Table S2: Genotyping Oligos Used in this Study, Related to STAR methods

Target	Name	5’ – 3’
Prdm1-cre	Blimp1-cre F	GCCGAGGTGCGCGTCAGTAC
	Blimp1-cre R	CTGAACATGTCCATCAGGTTCTTG
Trim28$^{f/f}$	Trim28 F	GGAATGGTTGTTCATTGGTG
	Trim28 R1 (No Excision)	ACCTTGGCCCATTTATTGATAAAG
	Trim28 R2 (Excision)	GCGAGCACGAATCAAGGTCAG
Sex	SMCX-1 (X)	CCGCTGCCAAATTCTTTGG
	SMCY-1 (Y)	TGAAGCTTTTGGCTTTGAG
Oct4-eGFP	Oct4-eGFP1	GATCACCTGGGGTTTGAGAA
	Oct4-eGFP2	CAAGGCAAGGGAGGTAGACA
	Oct4-eGFP3	AGGAACTGCTTCCTTCACGA

Table S3: Online Datasets Used in this Study, Related to STAR methods

Repository	Accession	SRA Record	Simple Name	Library Type
GEO	GSE60204	SRR1539456	D6_PGCLC_H3K27ac_Rep1	ChIP-seq
		SRR1539457	D6_PGCLC_H3K27ac_Rep2	ChIP-seq
		SRR1539503	D6_PGCLC_Input_Rep1	ChIP-seq
		SRR1539504	D6_PGCLC_Input_Rep2	ChIP-seq
GEO	GSE109770	SRR6519362	E10.5_PGC_Rep1	DNAse-seq
		SRR6519363	E10.5_PGC_Rep1	DNAse-seq
		SRR6519364	E12.5_PGC_XX_Rep1	DNAse-seq
		SRR6519365	E12.5_PGC_XX_Rep2	DNAse-seq
		SRR6519367	E12.5_PGC_XY_Rep1	DNAse-seq
		SRR6519368	E12.5_PGC_XY_Rep2	DNAse-seq
		SRR6519374	E14.5_PGC_XX_Rep1	DNAse-seq
		SRR6519375	E14.5_PGC_XX_Rep2	DNAse-seq
		SRR6519376	E14.5_PGC_XY_Rep1	DNAse-seq
		SRR6519377	E14.5_PGC_XY_Rep2	DNAse-seq
GEO	GSE141182	SRR10560100	E10.5_PGC_H3K9me3_Rep1	ChIP-seq
		SRR10560104	E10.5_PGC_H3K9me3_Rep2	ChIP-seq
		SRR13296472	E10.5_PGC_H3K9me3_Rep3	ChIP-seq
		SRR10560101	E10.5_PGC_Input_Rep1	ChIP-seq
		SRR10560105	E10.5_PGC_Input_Rep2	ChIP-seq
		SRR13296477	E10.5_PGC_Input_Rep3	ChIP-seq
		SRR10560108	E13.5_XX_PGC_H3K9me3_Rep1	ChIP-seq
		SRR10560114	E13.5_XX_PGC_H3K9me3_Rep2	ChIP-seq
		SRR13296474	E13.5_XX_PGC_H3K9me3_Rep3	ChIP-seq
		SRR10560106	E13.5_XX_PGC_Input_Rep1	ChIP-seq
		SRR10560112	E13.5_XX_PGC_Input_Rep2	ChIP-seq
		SRR13296479	E13.5_XX_PGC_Input_Rep3	ChIP-seq
		SRR10560111	E13.5_XY_PGC_H3K9me3_Rep1	ChIP-seq
		SRR10560117	E13.5_XY_PGC_H3K9me3_Rep2	ChIP-seq
		SRR13296476	E13.5_XY_PGC_H3K9me3_Rep3	ChIP-seq
		SRR10560109	E13.5_XY_PGC_Input_Rep1	ChIP-seq

		SRR10560115	E13.5_XY_PGC_Input_Rep2	ChIP-seq
		SRR13296481	E13.5_XY_PGC_Input_Rep3	ChIP-seq
DDBJ	DRA006633	DRR126197	E13.5_XY_PGC_H3K27ac_Rep1	ChIP-Seq
		DRR126198	E13.5_XY_PGC_H3K27ac_Rep2	ChIP-Seq
		DRR126199	E13.5_XY_PGC_Input	ChIP-Seq
		DRR126183	E13.5_XX_PGC_H3K27ac_Rep1	ChIP-Seq
		DRR126184	E13.5_XX_PGC_H3K27ac_Rep2	ChIP-Seq
		DRR126248	E13.5_XX_PGC_Input	ChIP-Seq
GEO	GSE118755	SRR7719557	E10.5_Gonadal_Soma_XY_Rep1	ATAC-Seq
		SRR7719558	E10.5_Gonadal_Soma_XY_Rep2	ATAC-Seq
		SRR7719555	E13.5_Gonadal_Soma_XY_Rep1	ATAC-Seq
		SRR7719556	E13.5_Gonadal_Soma_XY_Rep2	ATAC-Seq
		SRR7719559	E10.5_Gonadal_Soma_XX_Rep1	ATAC-Seq
		SRR7719560	E10.5_Gonadal_Soma_XX_Rep2	ATAC-Seq
		SRR7719553	E13.5_Gonadal_Soma_XX_Rep1	ATAC-Seq
		SRR7719554	E13.5_Gonadal_Soma_XX_Rep2	ATAC-Seq
GEO	GSE75751	SRR2980403	MERVL/Zscan4+ Rep 1	RNA-seq
		SRR2980404	MERVL/Zscan4+ Rep 2	RNA-seq
		SRR2980405	MERVL/Zscan4+ Rep 3	RNA-seq
		SRR2980406	E14 mESC NegativeControl_Rep1	RNA-seq
		SRR2980407	E14 mESC NegativeControl_Rep2	RNA-seq
		SRR2980408	E14 mESC NegativeControl_Rep3	RNA-seq
